# Serum- and glucocorticoid- inducible kinase 2, SGK2, is a novel autophagy regulator and modulates platinum drugs response in cancer cells

**DOI:** 10.1038/s41388-020-01433-6

**Published:** 2020-08-27

**Authors:** Valentina Ranzuglia, Ilaria Lorenzon, Ilenia Pellarin, Maura Sonego, Alessandra Dall’Acqua, Sara D’Andrea, Sara Lovisa, Ilenia Segatto, Michela Coan, Jerry Polesel, Diego Serraino, Patrizia Sabatelli, Paola Spessotto, Barbara Belletti, Gustavo Baldassarre, Monica Schiappacassi

**Affiliations:** 1 Division of Molecular Oncology, Centro di Riferimento Oncologico di Aviano (CRO), IRCCS, 33081 Aviano, Italy; 2 Epidemiology, Centro di Riferimento Oncologico di Aviano (CRO), IRCCS, 33081 Aviano, Italy; 3 Institute of Molecular Genetics, National Research Council, Bologna, Italy; 4 Rizzoli Orthopedic Institute, Bologna, Italy

**Keywords:** Cancer therapeutic resistance, Predictive markers

## Abstract

For many tumor types chemotherapy still represents the therapy of choice and many standard treatments are based on the use of platinum (PT) drugs. However, de novo or acquired resistance to platinum is frequent and leads to disease progression. In Epithelial Ovarian Cancer (EOC) patients, PT-resistant recurrences are very common and improving the response to treatment still represents an unmet clinical need. To identify new modulators of PT-sensitivity, we performed a loss-of-function screening targeting 680 genes potentially involved in the response of EOC cells to platinum. We found that SGK2 (Serum-and Glucocorticoid-inducible kinase 2) plays a key role in PT-response. We show here that EOC cells relay on the induction of autophagy to escape PT-induced death and that SGK2 inhibition increases PT sensitivity inducing a block in the autophagy cascade due to the impairment of lysosomal acidification. Mechanistically we demonstrate that SGK2 controls autophagy in a kinase-dependent manner by binding and inhibiting the V-ATPase proton pump. Accordingly, SGK2 phosphorylates the subunit V1H (ATP6V1H) of V-ATPase and silencing or chemical inhibition of SGK2, affects the normal autophagic flux and sensitizes EOC cells to platinum. Hence, we identified a new pathway that links autophagy to the survival of cancer cells under platinum treatment in which the druggable kinase SGK2 plays a central role. Our data suggest that blocking autophagy via SGK2 inhibition could represent a novel therapeutic strategy to improve patients’ response to platinum.

## Introduction

Platinum-based chemotherapy is employed in the treatment of a wide range of solid tumors, including head and neck, pancreas, colorectal, bladder, ovarian, and lung cancer [1, 2]. Despite a good rate of initial responses, platinum-based treatment often results in the development of chemoresistant recurrences leading to treatment failure [3–8]. This issue is particularly relevant for Epithelial Ovarian Cancer (EOC) where acquired resistance to platinum (PT) is a frequent event that predicts poor prognosis for patients [9, 10]. During the last years, several comprehensive genomic analyses of EOC have been performed to reveal new oncogenic drivers and possible mediators of PT-resistance in the most common histotypes [11–13]. These studies underscored the heterogeneity of EOC and the need of novel approaches to identify new possible therapeutic targets.

Many studies have been done to identify genes and mechanisms directly associated to resistance to PT therapy. PT-resistance has been linked to alterations in several processes such as drug transport, drug inactivation, DNA damage response, DNA repair, and apoptosis [14, 15]. Among general stress response pathways autophagy has also been associated to PT-resistance [16, 17]. In fact, autophagy has been shown to confer cancer cells the metabolic plasticity necessary to grow in suboptimal environments and to survive under therapy-induced stress [18–21].

We performed a loss-of function screening to identify genes able to impact on PT treatment targeting the pathways linked to PT-resistance in EOC. Using this approach, we unveiled SGK2, serum-and glucocorticoid- kinase 2, as a novel modulator of platinum sensitivity.

The SGK family is composed of three isoforms: SGK1, SGK2, and SGK3, belonging to the AGC kinase group [22, 23]. AGC family comprises 60 members, some of them extensively studied in cancer research such as AKT, S6K, and RSK. Literature data indicate that the three SGK isoforms share similar biochemical properties and structure, with 80% homology in their kinase domains, 44–68% in their carboxy termini non-catalytic domain and 25% at the NH2-terminus between SGK1 and SGK3 and almost no identity between SGK2 and the other isoforms [24]. Most of the scientific knowledge about SGK family roles in cellular physiology and in the development of human diseases is based on the study of SGK1 and SGK3 genes, while SGK2 is a virtually uncharacterized one. SGKs were initially identified as regulators of several transporters, channels, and pumps in the context of epithelial ions transport [25–28]. Recent works indicate that SGK1 and SGK3 have an emerging role in cancer biology sustaining tumor growth in presence of PI3K/AKT inhibition and suggest that these genes could have an oncogenic function [29–32].

In the current study, we have demonstrated a previously unrecognized role of SGK2 in PT-sensitivity exerted by the modulation of autophagic flux.

## Results

### SGK2 silencing sensitizes EOC cells to platinum

**Fig. 1:**
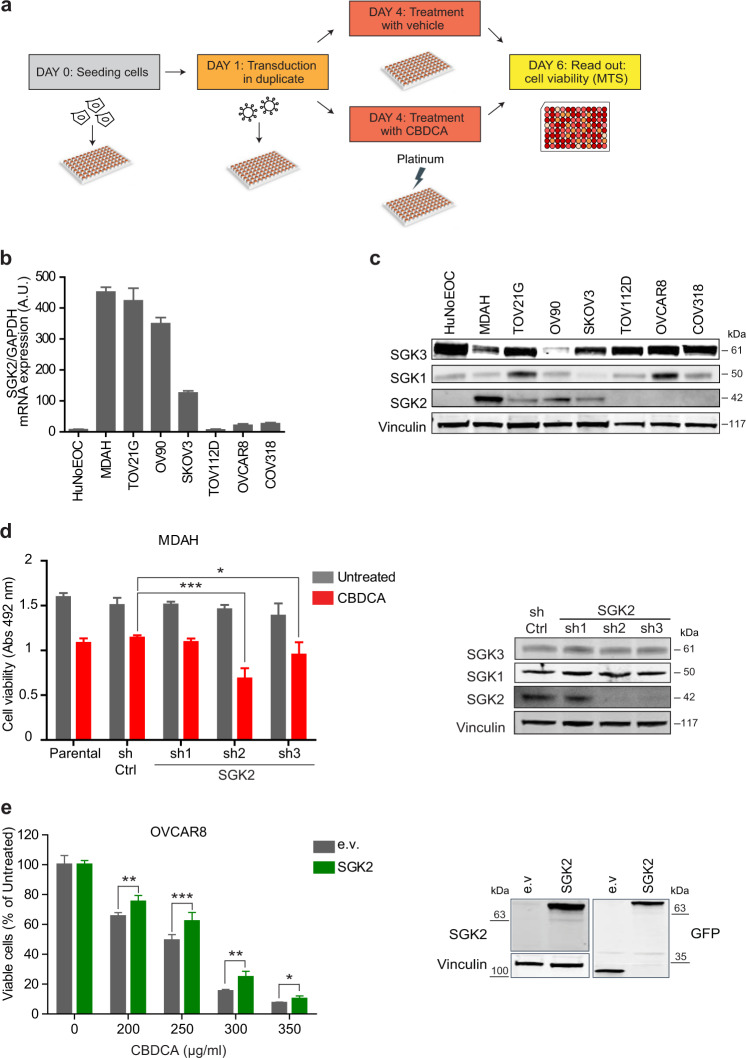
SGK2 silencing sensitizes ovarian cancer cells to platinum treatment. **a** Experimental design of the *loss-of-function* screening. Transduced cells were treated or not with CBDCA for 16 h using a dose able to induce only 10–20% of cell mortality. **b** SGK2 mRNA expression in the indicated EOC cell lines evaluated by qRT-PCR. **c** Western blot (WB) analyses evaluating SGK1, SGK2, and SGK3 expression in the indicated EOC cell lines. Vinculin was used as loading control. **d** Graph reports the viability of MDAH cells transduced with control (sh Ctrl) and three different SGK2 shRNAs, and then treated with CBDCA 140 µg/ml for 16 h as in **a**. On the right, WB analysis of SGK2, SGK1 and SGK3 expression in SGK2 silenced MDAH cells. **e** Graph reports the viability of OVCAR8 cells stably overexpressing EGFP-SGK2. Cells were treated with increasing doses of CBDCA and cell viability analyzed as in **d**. Results are expressed as percentage of CBDCA survived cells between treated and untreated cells (set as 100% as reference). On the right, WB analyses of SGK2 expression in the used cells. Vinculin was used as loading control. In **d** and **e** data represent the mean ± SD of three independent experiments. Significance was calculated using two-tailed, unpaired Student’s *t* test. ***p < 0.001, **p < 0.01, *p < 0.05. (See also Figs. S1 and S2).

To unveil key genes involved in the regulation of PT sensitivity of EOC cells we used a functional genomic approach targeting 680 genes related to apoptosis, p53 and DNA repair pathways with three shRNAs in two different EOC cell lines (MDAH-2774, herein MDAH, and SKOV3) (Fig. 1a and S1a). Statistical analysis and quality controls of this high-throughput screening identified 50 genes as possible PT-sensitizers (screening raw and analyzed data is reported in Data File S1 and Supplementary Materials and Methods). A subsequent screening using four different EOC cell lines was performed to validate these candidate genes (Data File S1, Section 8). Among the confirmed ones [33, 34] (described in Section 9, Data File S1), SGK2 silencing showed increased PT-induced death in three out of four different EOC cell lines used (i.e., MDAH, OV90 and SKOV3 but not in TOV112D) (Fig. S1b).

SGK2 expression (mRNA and protein) was analyzed in normal human epithelial ovarian cells (HuNoEOC) and in a panel of seven available EOC cell lines showing a good concordance between RNA and protein expression (Fig. 1b,c). Interestingly, SGK2 was not expressed in the TOV112D cell line (used in the validation screening) giving a possible explanation for the fact that SGK2 shRNAs did not affect PT-induced death in this cell line (Fig. S1b), although no correlation was observed between SGK2 expression and the PT-IC50 of the analyzed cells. To confirm the screening results, we used SGK2 expressing cells (MDAH, TOV21G, SKOV3, and OV90) and treated them with carboplatin (CBDCA). In all models SGK2 silencing significantly increased CBDCA-induced death, although some variability among the different shRNA was observed (Fig. 1d and S1c–e). Accordingly, overexpression of SGK2 in OVCAR8 cells (where endogenous SGK2 expression was barely detectable) increased the resistance to CBDCA (Fig. 1e). Moreover, SGK2 silencing in MDAH and TOV21G cells significantly decreased their CBDCA IC50 as evaluated by dose-response curves (Fig. S2a).

Given the high sequence homology described for the three members of the SGK family [24, 35] we also analyzed the expression of SGK1 and SGK3 in normal and cancer-derived EOC cells and tested if their silencing could have any role in PT-sensitivity. SGK3 was highly expressed in almost all tested cells while SGK1 was expressed in 6/7 tested EOC cell lines. Both proteins were easily detected in normal HuNoEOC (Fig. 1c). Using MDAH as a model, we observed that silencing of SGK3 and SGK1 was specific and did not affect their survival and the PT-sensitivity (Fig. S2b, c).

### SGK2 silencing sensitizes EOC cells to platinum via a kinase-dependent mechanism

**Fig. 2:**
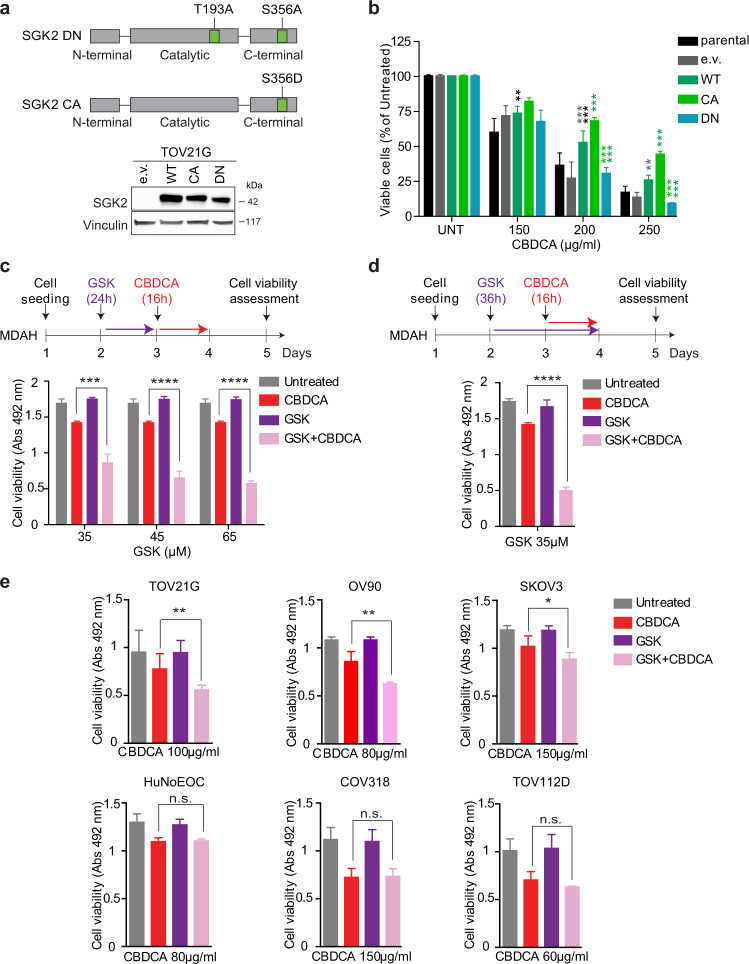
SGK2 silencing sensitizes ovarian cancer cells to platinum via a kinase-dependent mechanism. **a** Schematic representation of SGK2 dominant negative (DN) (pLPC-SGK2^T193A/S356A^) and constitutively active (CA) (pLPC-SGK2^S356D^) constructs (upper panel). Overexpression of the different SGK2 constructs in TOV21G cells was confirmed by WB (lower panel). Vinculin was used as loading control. **b** Graph reporting cell viability of TOV21G cells described in **a** and treated with increasing doses of CBDCA. Results are expressed as survival ratio (%) of CBDCA treated over untreated cells (set as 100% as reference) (e.v = empty vector). **c** Graph reporting cell viability of MDAH cells treated with the indicated doses of GSK650394 (GSK), followed by CBDCA 140 μg/ml as depicted in the experimental timeline shown above the graph. **d** Graph reporting cell viability of MDAH cells treated for 36 h with GSK 35 μM, in the presence of CBDCA 140 μg/ml for the last 16 h as depicted in the shown experimental timeline. **e** Graphs reporting cell viability of SGK2-expressing (TOV21G, OV90, and SKOV3) and SGK2 *not*-expressing cell lines (HuNoEOC, COV318, TOV112D) treated with GSK 35 μM, CBDCA at the indicated doses used alone or in combination using the timeline reported in **d**. Data represent the mean ± SD of three independent experiments. Significance was calculated using two-tailed, unpaired Student’s *t* test. ****p < 0.0001, ***p < 0.001, **p < 0.01, ns not significant. (See also Fig. S3).

To understand how SGK2 regulates the PT-sensitivity in EOC cells, we first investigated if its kinase activity was involved. To this aim, we generated a SGK2 kinase-dead (KD) mutant by substituting the Lys64 residue within the ATP binding region with a methionine, as described by others [28]. This mutant had no effect on PT-sensitivity in TOV21G cells (Fig. S3a), suggesting a possible role for SGK2 kinase activity on PT-induced death. Based on the work of Hemmings and collaborators demonstrating that mutation of Thr256 and Ser422 in alanine confers to SGK1 a dominant-negative (DN) activity [36], we next generated a SGK2 DN construct by substituting with an alanine the corresponding Thr193 and Ser356 residues in the catalytic and activating domain (Fig. 2a). We also substituted Ser 356 in the activating domain with the phosphomimetic residue Asp (S356D) to generate a SGK2 constitutively active (CA) mutant. In dose-response experiments, the overexpression of SGK2 DN increased and SGK2 CA decreased the sensitivity of TOV21G cells to CBDCA (Fig. 2b). Taken together these data support the possibility that SGK2 kinase activity plays a role in the control of PT-induced death. Accordingly, pretreatment of EOC cells for 24 h with increasing doses (from 35 to 65 µM) of the small-molecule GSK650394, a specific SGK1/SGK2 inhibitor [37] significantly increased MDAH cells sensitivity to PT showing no toxic effects when used alone (Fig. 2c). This effect was amplified when GSK650394 was maintained in culture with CBDCA for a total time of 36 h, again with no appreciable toxicity (Fig. 2d). A similar increase in PT-sensitivity was observed using cisplatin (CDDP) in place of CBDCA (Fig. S3b). Accordingly, GSK650394 significantly decreased CBDCA IC50 in dose-response analyses performed in MDAH and TOV 21G but not in COV318 cells that do not express SGK2 (Fig. S3c, d). Using this approach, we identified the 35 μM dose as the most effective to be used in combination with CBDCA in all tested cells (Fig. S3c). Next, by using the combined GSK650394+CBDCA treatment on a panel of EOC cells we verified that GSK650394 increased PT-sensitivity in SGK2 expressing cells (MDAH, TOV21G, OV90, and SKOV3) but not in those that did not express appreciable levels of SGK2 protein (COV318, TOV112D, and normal HuNoEOC) (Fig. 2e). Similarly, GSK650394 did not have any effect on PT-sensitivity in TOV21G cells stably expressing SGK2 DN (Fig. S3e). These results indicated that the GSK650394 inhibitor sensitized EOC cells to CBDCA in a SGK2-dependent manner and confirmed the role of SGK2 kinase activity in the control of PT-sensitivity.

### SGK2 inhibition also improves platinum response in platinum-resistant EOC cells, primary EOC cells and breast and head and neck cancer cells

**Fig. 3:**
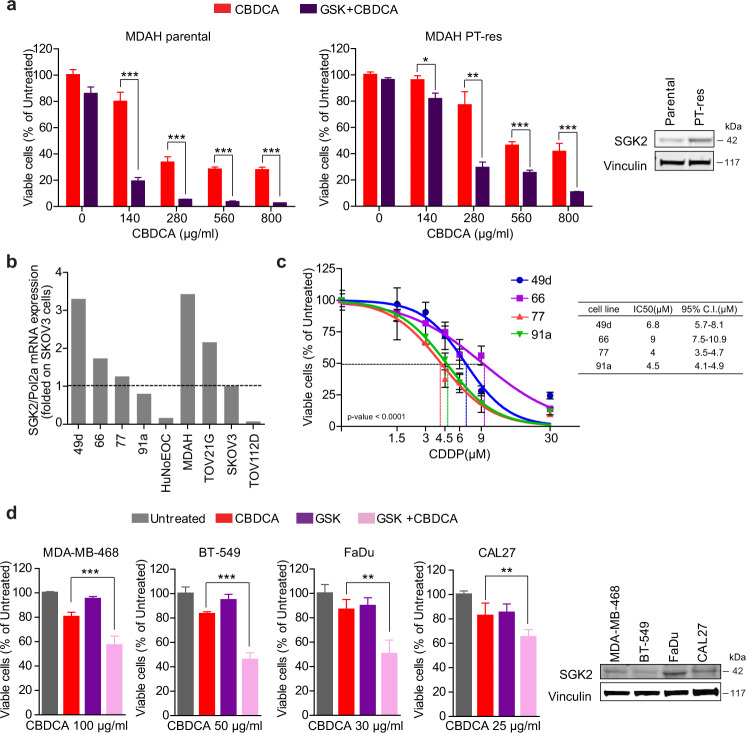
SGK2 inhibition improves platinum response in platinum-resistant EOC, primary EOC, head and neck squamous cell carcinoma and breast cancer cells. **a** Graph reporting cell viability of MDAH parental and PT-res cells treated with GSK650394 35 µM for 24 h (GSK), in the presence of increasing doses of CBDCA for additional 16 h. Results are expressed as survival ratio (%) between treated and untreated cells (set as 100% as reference). On the right, WB analysis reports the expression of SGK2. Vinculin was used as loading control. **b** SGK2 mRNA expression in the indicated primary and established EOC cell lines evaluated by qRT-PCR normalized to housekeeping gene POL2A and then folded on SKOV3 SGK2 expression. **c** Nonlinear regression analyses of cell viability assay in primary EOC cells treated with increasing doses of CDDP for 16 h. Results are expressed as percentage of viable cells respect to the untreated cells and represent the mean (±SD) of three biological replicates. The table on the right reports the IC50 and the confidence interval (C.I.) of each condition. Fisher’s exact test was used to calculate the global *p* value reported in the graph. **d** Graph reporting cell viability of MDA-MB-468, BT-549 (TNBC cell lines) and FaDu and CAL27 (HNSCC cell lines) treated with GSK650394 35 µM (GSK) and CBDCA as indicated. Results are expressed as survival ratio (%) between treated and untreated cells (set as 100% as reference). On the right, WB analysis reporting the expression of SGK2. Vinculin was used as loading control. (See also Fig. S4).

We next explored if SGK2 inhibition could re-sensitize platinum-resistant cells (PT-res) to platinum. To this aim we exploited the isogenic MDAH PT-res cells recently generated in our lab (herein MDAH PT-res) [38]. MDAH PT-res cells had higher levels of SGK2 and the combined GSK650394 + CBDCA treatment induced an increase in MDAH PT-res cell death when compared to CBDCA single treatment at the tested doses (Fig. 3a).

Then we analyzed SGK2 expression and PT-sensitivity in primary cells isolated in our lab from EOC patients’ surgical samples. We analyzed four different primary cell lines (Fig. S4) comparing SGK2 mRNA expression to EOC cell lines, setting as cut off value the expression of SGK2 in the SKOV3 cells (which had the lowest SGK2 expression detectable by western blot, Figs. 1c and 3b). The 49d and 66 primary EOC cells that displayed the highest SGK2 expression also had the highest CDDP IC50 (Fig. 3c), supporting a possible correlation between SGK2 expression and response to PT in primary cultures.

Moreover, the GSK650394+CBDCA treatment increased cell death also in Triple-Negative Breast Cancer (TNBC) (MDA-MB-468 and BT-549) and in Head and Neck Squamous Cancer (HNSCC) (FaDu and CAL27) cells (Fig. 3d), two human cancer types known to be treatable with PT in the clinical practice.

### SGK2 modulates autophagic flux

**Fig. 4:**
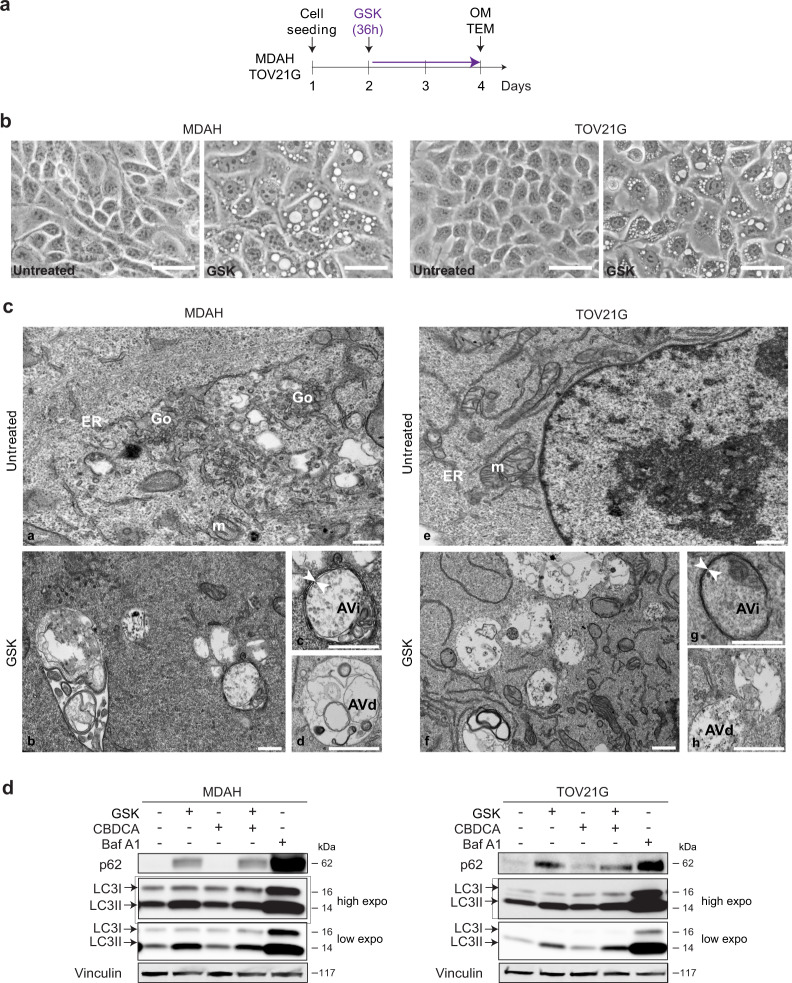
SGK2 inhibition alters autophagic flux. **a** Experimental timeline of cells studied with optical (OM) and transmission electron microscopy (TEM). MDAH and TOV21G cells were treated with GSK650394 (GSK) 35 µM for 36 h. **b** Representative optical microscopy pictures of MDAH and TOV21G cells treated or not with GSK650394 (GSK) as reported in **a**. Cytoplasmic vacuoles are present only in GSK650394 (GSK) treated cells. Scale bars = 50 µm. **c** TEM ultrastructure analyses of MDAH (left panels) and TOV21G (right panels) cells. **a** and **e** Untreated cells; **b** and **f** GSK650394-treated cells (GSK) showing cytoplasmic vacuoles accumulation; **c** and **g** Magnification of initial autophagic vacuoles (AVi) (white arrowheads indicate double-membrane) and degradative autophagic vacuoles (AVd) vacuoles (**d** and **h**). Highlighted intracellular organelles in untreated cells are endoplasmic reticulum (ER), Mitochondrion (m) and Golgi apparatus (Go). Scale bar: 300 nm. **d** Western blot analyses evaluating the expression of the autophagy markers LC3I/LC3II and p62 in MDAH and TOV21G cells, treated with GSK650394 35 μM (36 h), CBDCA 140 μg/ml (16 h) and GSK+CBDCA (as in Fig. 2d) compared to the effect of Bafilomycin A1 (Baf A1). Vinculin was used as loading control. (See also Figs. S5 and S6).

In performing the experiments described above, we observed that GSK650394 induced the formation of cytoplasmic vesicles in EOC cells both when used as single treatment (Fig. 4a, b) and in combination with CBDCA (Fig. S5a, b). This vesicles’ formation was noticed only in the SGK2-expressing cell lines (MDAH and TOV21G) but not in TOV112D cells that did not express SGK2 (Fig. S5b). Vesicles’ formation was reversible since the withdrawal of GSK650394 from the medium resulted in their disappearance within 24 h (Fig. S5c). The direct involvement of SGK2 in the formation of these cytoplasmic vesicles was confirmed by silencing SGK2 in MDAH and TOV21G cells (Fig. S5d), although less pronounced than the one observed with GSK650394. Similar results were recapitulated in all tested TNBC and HNSCC cell lines (Fig. S5e, f).

Transmission Electron Microscopy (TEM) in MDAH and TOV21G cells was next used for a deeper understanding of the nature of these cytoplasmic vesicles. TEM showed that vesicles induced by GSK650394 treatment had an ultrastructure compatible with that of autophagic vacuoles (AV) [39, 40] with typical features of initial autophagic vacuoles (AVi), that could be mostly categorized as autophagosomes (AP) as indicated by the presence of double membrane (Figs. 4a, c and S6a). APs were present in approximately 10% of GSK650394-treated cells. Moreover, almost all treated cells also presented degradative autophagic vacuoles (AVd) accumulating partially degraded material (Fig. 4c). Neither AVi nor AVd were observed in untreated cells. These data suggested that GSK650394 treatment resulted in the alteration of the autophagic flux likely by affecting the rate at which the autophagy machinery identifies, segregates and disposes of substrates through lysosomal degradation therefore leading to the accumulation of AV [41–43].

To confirm and better characterize the link between GSK650394 treatment and autophagy, we first tested the expression of two specific markers of autophagy namely p62/SQSTM1 (herein p62) and LC3 in our experimental model. As a positive control we used Bafilomycin A1 (Baf A1), a known inhibitor of the autophagy flux at late stages that, as expected, increased PT-sensitivity of MDAH cells (Figs. 4d and S6b). Western blot analyses showed that GSK650394 treatment, alone or in combination with platinum, induced a clear increase in p62 levels, a change in the ratio between unlipidated (cytosolic) and lipidated (membrane bound) forms of LC3 (LC3I/LC3II ratio) and an overall increase of LC3 forms, in both MDAH and TOV21G cells comparable to the effects exerted by Baf A1 (Figs. 4d and S6c). Similar observations were made in TNBC and HNSCC cells where GSK650394 treatment also induced the increase of p62 and LC3II (Fig. S6d). Increased levels of p62 and of LC3 lipidation (LC3II) together with the accumulation of AV, are possible markers of autophagy inhibition [42, 44, 45], overall confirming the possibility that SGK2 is a previously undisclosed regulator of autophagy.

### SGK2 inhibition alters acidification of vacuolar cellular compartment

**Fig. 5:**
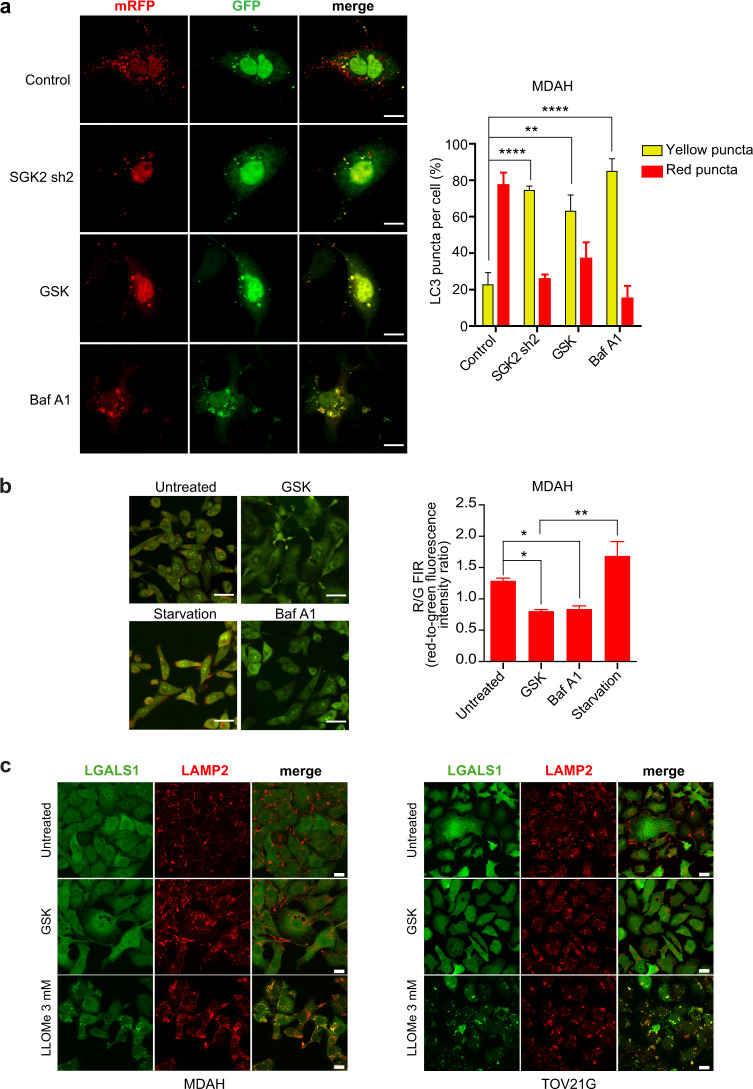
SGK2 inhibition alters acidification of vacuolar cellular compartment. **a** Representative pictures of confocal microscopy analyses of MDAH cells transfected with mRFP-GFP-LC3 untreated (Control), treated with GSK650394 35 μM for 36 h (GSK) or with Bafilomycin A1 0.2 μM for 30 min (Baf A1) or transduced with SGK2 shRNA (SGK2 sh2) (scale bar: 12 μm). The graph on the right reports the percentage of yellow/red LC3 puncta counted per cell (number of cells/experimental condition = 15–20). **b** Representative pictures of confocal microscopy analyses of MDAH cells treated or not with GSK650394 35 μM (GSK) for 36 h and then stained with acridine orange. Cells serum-starved for 4 h (Starvation) or treated with Bafilomycin A1 0.2 μM for 30 min (Baf A1) were used as positive and negative controls, respectively (scale bar: 18 μm). On the right, the graph reports the average red-to-green fluorescence intensity ratio (number of fields evaluated per condition = 8–10). **c** Representative pictures of confocal microscopy analyses for the evaluation of LGALS1 (galectin-1, green) and LAMP2 (red) staining of MDAH (left) and TOV21G (right) cells treated or not with GSK650394 35 μM (GSK) or LLOMe 3 mM (used as positive control of LMP (Lysosomal Membrane Permeabilization)) and then evaluating LGALS1 (galectin-1, green) (Scale bar: 20 μm).

To better define how SGK2 down-modulation could alter the autophagic flux, we exploited a fluorescence assay using a LC3 construct tagged to mRFP-GFP tandem fluorescent proteins (ptfLC3) [46]. The use of ptfLC3 allows monitoring vesicles maturation since the EGFP protein is quenched in acidic environments (such as lysosomes and autolysosomes), whereas mRFP is more stable under acidic conditions allowing defining if autophagic vesicles are (red fluorescence) or not (red plus green = yellow fluorescence) fused with lysosomes [42, 46, 47]. Using this tool in MDAH cells we observed that exposure of cells to GSK650394 or SGK2 silencing inverted the ratio between red and yellow LC3 positive vesicles, here referred to as red or yellow puncta (Fig. 5a). Similarly, Baf A1 treatment that blocks lysosome acidification, also resulted in a strong increase in the number of yellow puncta. These data suggested that SGK2 inhibition could suppress autophagy at a late stage likely blocking the fusion of autophagosome to lysosome and/or autolysosome acidification.

These data were confirmed using the acridine orange (AO) staining. AO is an acidotropic dye that turns from green fluorescence when located in the cytosol to a granular red fluorescence when included into acidic compartments [42, 48]. AO staining is modified by serum starvation (increased autophagy, with increased Red/Green fluorescence intensity ratio (R/G FIR)) and Baf A1 (blocked autophagy with decreased R/G FIR). Using these two treatments as positive and negative controls, we observed that GSK650394 and Baf A1 induced a strong reduction of R/G FIR while serum starvation increased the granular R/G FIR when compared to untreated MDAH cells (Fig. 5b). Since the reduction of AO R/G FIR could also be due to lysosomal membrane permeabilization (LMP) we tested if GSK650394 affected LMP rather than autophagosome maturation/acidification using the so-called galectin assay and the L-leucyl-L-leucine methyl ester (LLOMe) as positive control. LLOMe is an inducer of LMP that results in exposure of N-acetyl-lactosamine-rich carbohydrate shield on the lysosomal membrane that therefore is bound by the translocation of LGALS1 (galectin-1). As a consequence, the LGALS1 staining changes from diffuse to punctate upon LMP induction. Data obtained in both MDAH and TOV21G showed that LLOMe but not GSK650394 induced the appearance of LGALS1 punctate staining that also co-localize with the lysosomal marker LAMP2 (Fig. 5c), overall confirming that SGK2 inhibition did not directly induce LMP.

**Fig. 6:**
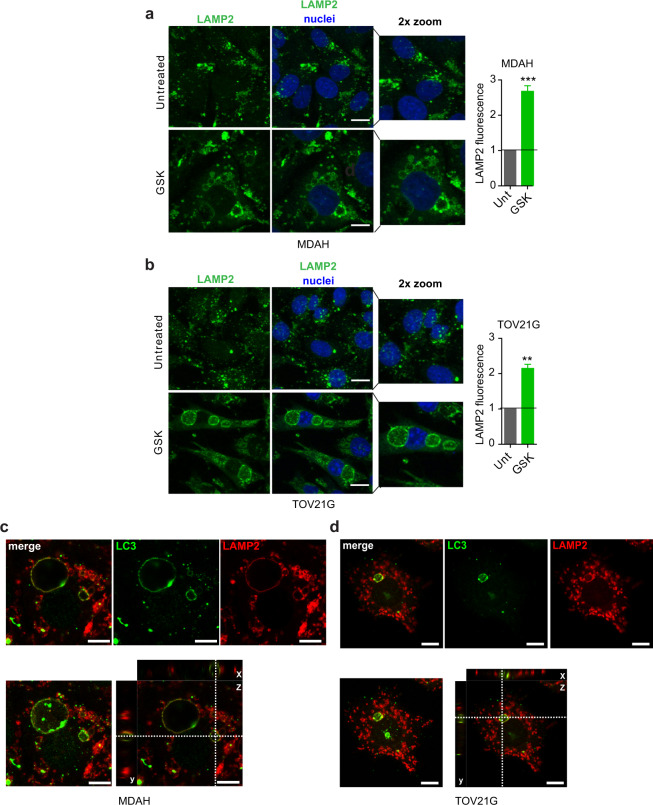
SGK2 inhibition induces the accumulation of autophagolysosomes. Representative pictures of confocal microscopy immunofluorescence analyses evaluating the expression and localization of LAMP2 in untreated or GSK650394 (GSK)-treated MDAH **a** and TOV21G **b** cells. A zoomed area is reported for each condition. Nuclei were stained with propidium iodide and pseudo colored in blue (scale bars: 9 μm). Graphs on the right report the average LAMP2 fluorescence intensity per field (average number of cells evaluated per condition = 90 cells). Results are expressed as fold of green fluorescence respect to untreated. Representative pictures of confocal microscopy immunofluorescence analyses evaluating LC3 (green) and LAMP2 (red) expression and localization in MDAH **c** and TOV21G **d** cells treated with GSK650394. Upper panel shows a representative best focus plane with single and merged channels, as indicated. Lower panel shows the maximum projection (left) and the xyz projections (right) showing the co-immunostaining of LC3 and LAMP2 in a vacuole after GSK650394 treatment. Scale bar:10 μm. In the figure data are mean ± SD. Significance was calculated using two-tailed, unpaired Student’s *t* test. ***p < 0.001, **p < 0.01. (See also Fig. S7).

Our data so far indicated that the inhibition of SGK2 kinase activity results in the block of autophagy at the stage of autophagosome/lysosome fusion and/or autolysosome acidification. To confirm these data, we stained control and GSK650394-treated cells (MDAH and TOV21G) with LAMP2 (Lysosomal Associated Membrane Protein 2) and observed a clear upregulation of LAMP2 staining in treated cells (Fig. 6a, b). LC3/LAMP2 co-staining revealed that both these molecules were present on the membrane of the large vacuoles observed under GSK treatment in both MDAH and TOV21G cells (Fig. 6c, d). Finally, co-staining of p62 with LAMP2 or SGK2 demonstrated that both LAMP2 and SGK2 co-localized on the autophagic vacuoles’ membrane in GSK650394-treated cells (Fig. S7a, b). Overall, the collected data suggested that SGK2 inhibition induced the accumulation of AVd with changes in acidic cellular compartment but no effect on autophagosome/lysosome fusion or lysosome permeability.

### SGK2 inhibition impairs lysosomal functionality

**Fig. 7:**
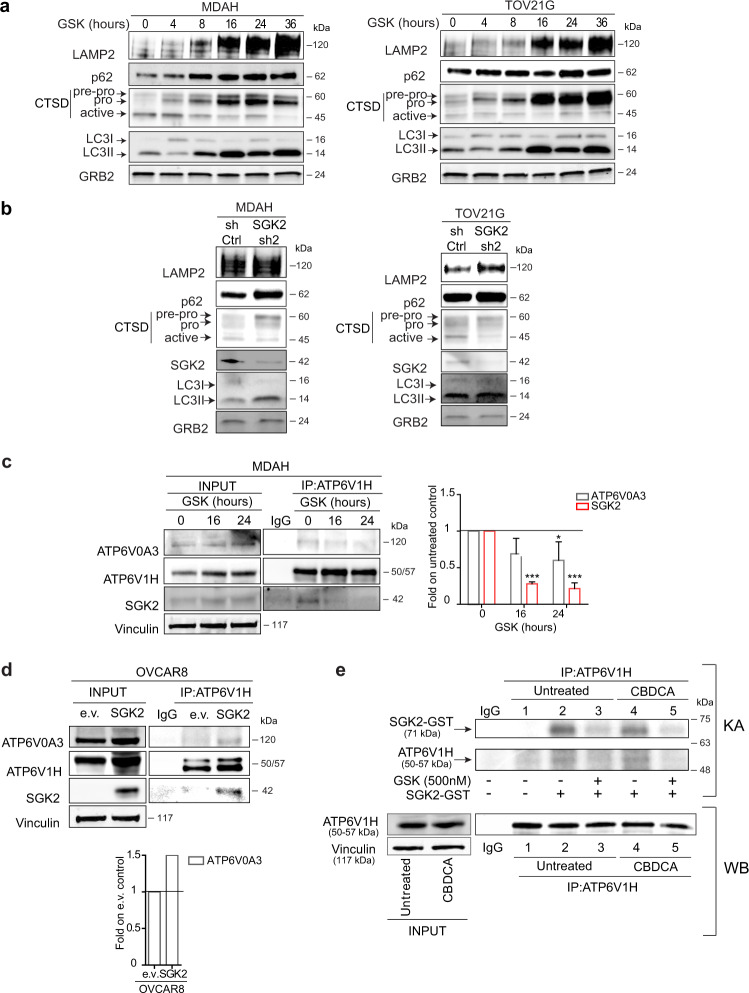
SGK2 inhibition causes autophagy blockade altering activation of lysosomal cathepsins. WB analyses of LAMP2, p62, Cathepsin D (CTSD) and LC3I/LC3II in cell lysates from MDAH and TOV21G cells treated with GSK650394 35 μM (GSK) for the indicated time **a** or silenced for SGK2 expression **b**. GRB2 was used as loading control. **c** Co-immunoprecipitation (IP) analysis of endogenous ATP6V1H with SGK2 and ATP6V0A3 in MDAH cells untreated or treated with GSK650394 35 μM (GSK) for 16 and 24 h. Cell lysates immunoprecipitated with ATP6V1H were probed for the expression of ATP6V1H, SGK2, and ATP6V0A3. The expression of the three proteins in the corresponding lysates (INPUT) is reported on the left. The right graph shows the quantification of SGK2 and ATP6V0A3 bound to ATP6V1H in GSK treated cells expressed as fold respect to untreated cells and represents the mean ± SD of three independent experiments. Significance was calculated using two-tailed, unpaired Student’s *t* test. ***p < 0.001, *p < 0.05. **d** Co-immunoprecipitation (IP) analysis of endogenous ATP6V1H with SGK2 and ATP6V0A3 in OVCAR8 cells stably overexpressing SGK2. Cell lysates immunoprecipitated with ATP6V1H were probed for the expression of ATP6V1H, SGK2 and ATP6V0A3. The expression of the three proteins in the corresponding lysates (INPUT) is reported. The lower graph shows the quantification of ATP6V0A3 bound to ATP6V1H in SGK2 overexpressing cells expressed as fold respect to cells transfected with the empty vector (e.v.). **e** In vitro kinase assay (upper panel) using SGK2 active recombinant protein incubated with endogenous ATP6V1H immunoprecipitated from untreated and CBDCA-treated MDAH cells (500 μg/ml 16 h) (lower panel). Western blot analyses of 1/10 of the immunoprecipitated ATP6V1H protein used in each line (1–5) and the corresponding lysates (INPUT) are reported (lower panel). Vinculin was used as loading control in **c**–**e**. IgG indicates a lysate IP with unrelated antibody. (See also Fig. S8).

We next explored from a biochemical point of view if SGK2 inhibition could impact on lysosomal activity. Time-course analysis of GSK650394-treated MDAH and TOV21G cells clearly revealed that the activation of the lysosomal proteases cathepsin-D, -L and -B was impaired in a time-dependent manner resulting in the accumulation of their *non*-active forms (Figs. 7a and S8a). The accumulation of inactive cathepsins was accompanied by the upregulation of LAMP2, p62, and LC3II (Fig. 7a). The molecular changes induced by GSK650394 were mostly recapitulated in SGK2-silenced cells although the modifications were less evident (Fig. 7b). Finally, over-expression of SGK2 in OVCAR8 cells reduced the expression pattern of p62 and LC3II and increased the expression of cathepsin D (Fig. S8b).

### SGK2 binds and phosphorylates the V-ATPase proton pump

All the biochemical and biological collected results (Figs. 5–7) suggested that SGK2 genetic or pharmacological inhibition impaired autolysosome acidification and degradation capacity with the consequent accumulation of late autophagic vacuoles (AVd). Since lysosome acidification depends on the activity of vacuolar H^+^-ATPase proton pump (V-ATPase) [49] and SGK proteins could regulate different ATPases [50, 51], we hypothesized that inhibition of SGK2 could alter the activity of this proton pump. In silico analyses predicted that SGK2 could phosphorylate several Serine and Threonine residues in the V1H and V0A3 subunits which belong, respectively, to the V1 and V0 domains of V-ATPase (Fig. S8c). We explored this possibility by testing if SGK2 could interact with any of these subunits. By immunoprecipitation analysis we observed that endogenous SGK2 co-precipitated with ATP6V1H and that this interaction was impaired by GSK650394 inhibitor treatment in a time-dependent manner (Figs. 7c and S8d). In addition, GSK650394 treatment affected the ATPase complex since it also impaired the co-precipitation of ATP6V0A3 with ATP6V1H. These observations were confirmed by over-expressing SGK2 in OVCAR8 cells where SGK2 co-precipitated with ATP6V1H and ATP6V0A3 subunits and increased the association between the two V-ATPase subunits (Fig. 7d). These evidences indicated that SGK2 interaction with V-ATPase could contribute directly to its activity, since the reversible assembly/disassembly of its V0 and V1 sectors represents an important regulatory mechanism of V-ATPase pump functionality [52, 53]. Next we tried to verify if SGK2 could phosphorylate the immunoprecipitated ATP6V1H subunit using recombinant SGK2 protein in in vitro kinase assays (Fig. 7e). These assays revealed that SGK2 phosphorylated in vitro ATP6V1H subunit immunoprecipitated from both untreated and platinum treated EOC cells. Importantly, the GSK650394 inhibitor impaired the SGK2-dependent phosphorylation of ATP6V1H (Fig. 7e). It is interesting to note that SGK2 underwent a specific auto-phosphorylation (Figs. 7e and S8e) that, in our knowledge, was not previously described. This event was pointed out by the presence of a radioactive signal also when SGK2-GST was incubated only with γP32ATP in vitro while it did not phosphorylate a GST-Rb fragment used as a control (Fig. S8e).

### SGK2 is involved in platinum-induced autophagy response in EOC cells

**Fig. 8:**
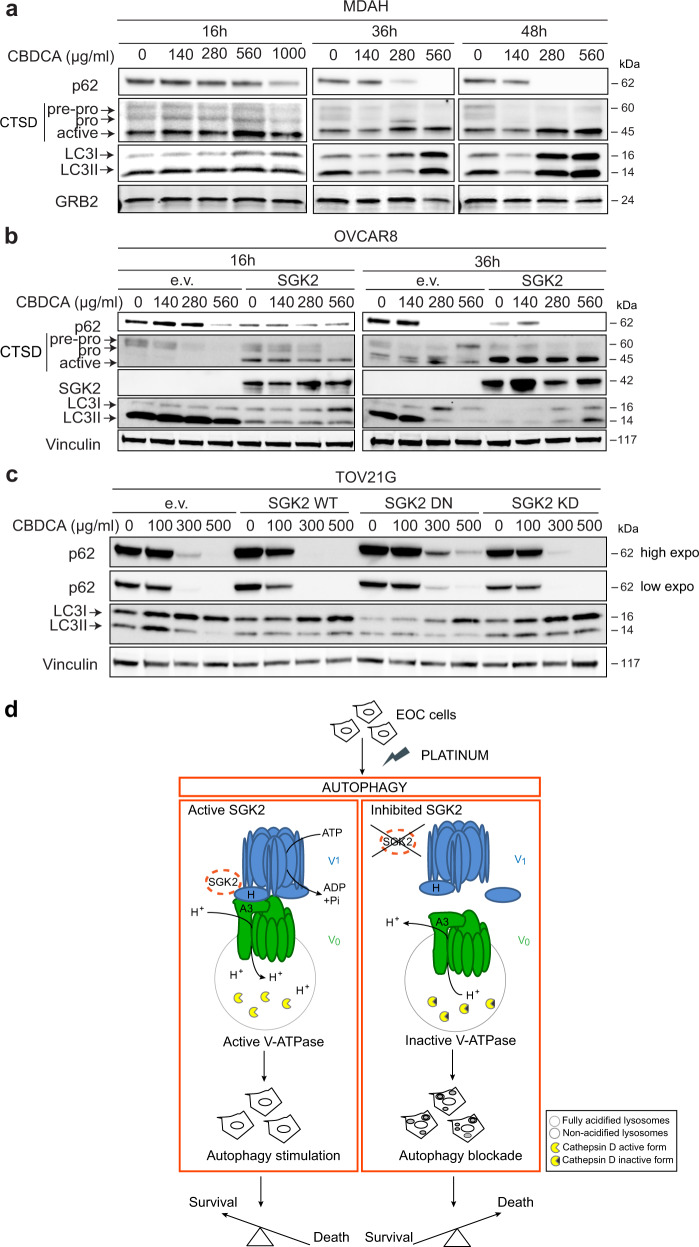
Platinum treatment stimulates autophagy in EOC cells. WB analyses of p62, Cathepsin D (CTSD) and LC3I/LC3II expression in MDAH cells **a** or in control and SGK2-overexpressing OVCAR8 cells **b** treated with increasing doses of CBDCA for the indicated times. **c** WB analysis of autophagy markers in TOV21G cells overexpressing SGK2 WT, DN and KD mutants treated as in **a** and **b** for 16 h. GRB2 **a** or Vinculin **b**, **c** were used as loading control. **d** Schematic representation of the possible role of SGK2 role in the regulation of platinum sensitivity of EOC cells. Platinum treatment induces an autophagic response as a pro-survival pathway in which SGK2 acts as an autophagy stimulator. In SGK2 expressing cells, SGK2 by interacting with the lysosomal V-ATPase proton pump, ensures the maintenance of the correct acid pH necessary for the activation/maturation of lysosomal enzymes (left panel). Silencing or chemical inhibition of SGK2 results in V-ATPase disassembly, impaired lysosomal degradation capacity and the consequent inhibition of autophagic flux with accumulation of giant autophagolysosomes (depicted inside the cell, right panel). Autophagy inhibition eventually results in an increased EOC cells sensitivity to platinum treatment and enhanced cell death (right panel).

The collected data suggested that SGK2 controls autophagic flux by ensuring the correct lysosomal acidification. Autophagy has been described as a pro-survival response to chemotherapeutic drugs in cancer cells, and impairment of autophagy could represent a novel therapeutic strategy to improve the chemotherapy efficacy and/or overcome the onset of chemotherapy resistance [54–58]. To verify if autophagy was implicated in the PT-response in the used models, we first treated MDAH cells with increasing doses of CBDCA for up to 48 h and observed a time- and dose-dependent stimulation of autophagy characterized by increased p62 clearance, LC3 I/II ratio and cathepsin D active form (Fig. 8a). Interestingly, CBDCA induction of autophagy, revealed by decreased expression of p62 in western blot analyses, was also confirmed in breast and head and neck cancer cells (Fig S9a).

We also explored autophagy and apoptosis markers 24 and 36 h after treatment withdrawn that represent the timing at which we observed increased cell death by the GSK650394+CBDCA (release in Fig S9b). In complete agreement with the biological data we observed that in cells treated with GSK650394+CBDCA modulation of autophagy preceded the appearance of apoptotic markers (i.e., cleaved PARP-1 and caspase-9), suggesting that the combination treatment induced increased cell death by impairing autophagy with a consequent induction of apoptosis.

To better dissect molecular events, we observed that stable over-expression of SGK2 in OVCAR8 cells that resulted in increased PT-resistance (Fig. 1e) also increased autophagy as demonstrated by the higher clearance of p62, inversion of LC3 I/II ratio and by a marked increase in active cathepsin D (Fig. 8b).

Next, by overexpressing SGK2 WT, KD and DN constructs in TOV21G we observed that the WT protein induced a rapid down modulation of p62 and LC3 already at the low dose of 100 μg/ml CBDCA while the DN mutant induced a persistent expression of p62 likely due to autophagy blockade (Fig. 8c). As expected, the expression of SGK2 KD did not induce any significant difference respect to TOV21G parental cells transfected with empty vector (Fig. 8c).

The data collected so far demonstrated that in EOC cells CBDCA treatment induced autophagy as a surviving mechanism sustained by SGK2 kinase activity. Accordingly, by treating MDAH cells with increasing doses of CBDCA in presence or not of GSK we observed a reversion of CBDCA-induced autophagy in cells treated with GSK650394+CBDCA. This reversion was evidenced by both delayed p62 clearance and inversion ratio LC3I/II (Fig. S10a).

We next asked if induction of autophagy was commonly induced by other chemotherapy used in the treatment of EOC and tested the effects of Taxol on p62 and LC3 expression in MDAH cells. Dose and time response curve showed that Taxol did not elicit any modification in their expression over a 36 h period (Fig. S10b). Accordingly, SGK2 inhibition by GSK650394 did not affect Taxol induced cell death in MDAH cells (Fig. S10c), overall supporting the hypothesis that SGK2 kinase activity protects cancer cells from PT-induced death by regulating autophagy.

Finally, by interrogating the Oncomine [59, 60] (http://www.oncomine.org) datasets we found that SGK2 is not only overexpressed but also amplified in a subset of EOC compared to normal tissue (Fig. S11a–c), suggesting that it could play a role in the human pathology.

## Discussion

Starting from an unbiased loss-of-function screening that identified SGK2 as a druggable modulator of PT sensitivity in EOC cells, we characterized the role of this under-studied kinase in the control of autophagy, an escaping strategy activated by cancer cells to survive under PT treatment. We demonstrated that genetic or pharmacological inhibition of SGK2 could block the induction of the autophagic process stimulated by PT, eventually increasing PT-induced cell death, in vitro.

It is known that autophagic responses in cancer cells operate in a highly dynamic network that affects complex processes going beyond intracellular homeostasis maintenance [57]. In this sense, the autophagic network plays paradoxical context-dependent roles being involved in tumor suppression and tumor promotion [58, 61]. Several evidences pointed out a potential role of autophagy in the ability of cancers to develop resistance to chemotherapy, suggesting a major impact of autophagy in the response to treatment [62, 63]. Here we report that in EOC cells chemotherapy with diverse mechanisms of action (CBDCA vs Taxol) differently impacted on autophagy induction, further confirming the context-dependent role of autophagy in cancer.

Autophagy is a highly conserved dynamic catabolic process that involves the formation of double membrane vesicles known as autophagosomes that engulf cellular proteins and organelles for delivery to the lysosome. When the autophagosome fuses with the lysosome, the content is degraded and macromolecular precursors are recycled or used to fuel metabolic pathways [55]. The adapter protein p62, which targets specific substrates to autophagosomes, and LC3II are degraded along with other cargo proteins and can be used as a measure of autophagic flux. The appearance of more autophagic elements (i.e., autophagosomes or autolysosomes) does not necessarily mean more autophagy. In fact, in many cases autophagosomes may accumulate due to a block in trafficking to lysosomes without any change in autophagosome biogenesis, whereas an increase in autolysosomes may indicate a reduction in degradation activity. In this sense, lysosomal digestion is a stage of autophagy and analyzing its proficiency is a fundamental part of the evaluation of autophagic flux or complete autophagy [42]. Lysosomes are characterized by highly acidic pH that provides optimal condition for the activity of its proteolytic enzymes like cathepsins [49]. Lysosomal pH gradient is mainly generated and maintained by V-ATPase, a multisubunit protein complex that hydrolyzes ATP to pump protons into the lysosome [52, 53].

In this context our data suggest that SGK2 genetic or pharmacological inhibition induces an accumulation of autophagosomes, in particular of giant autolysosomes, that was accompanied by an accumulation of inactive cathepsins [64]. We propose that SGK2 kinase activity has a role in the activation of V-ATPase favoring the V0V1 sectors assembly of this proton pump, likely via phosphorylation of the ATP6V1H subunit. We observed that SGK2 is able to phosphorylate ATP6V1H in in vitro kinase assays, (Fig. 7c) and, hypothetically, that this could also happen in a cellular context since GSK650394 treatment on MDAH cells impaired SGK2-ATP6V1H/V0A3 interaction. However, further experiments should confirm this possibility. Our evidences also support the possibility that SGK2 inhibition promotes ATPase disassembly, reducing its activity in maintaining acidic lysosomal pH for optimal proteases maturation/activity. The impaired activation of cathepsins subsequent to SGK2 inhibition, then reduces lysosomal degradation capacity with the consequent accumulation of autolysosome and resulting inhibition of the autophagic flux (Fig. 8d). It is worth noting that pharmacological inhibition of SGK2 with GSK650394 better blocks the autophagic flux than the silencing of the gene. This partial discrepancy could be due to the incomplete knock out of the SGK2 protein achievable with the use of the shRNAs technology or possible off target effect of the GSK650394 molecule. Future work with different molecular approaches could help in dissecting these two possibilities.

In line with our results, recent works reported that silencing different subunits of V-ATPase proton pump enhances cytotoxicity of paclitaxel and platinum drugs in cervical and ovarian cancer, respectively [65, 66]. These evidences supported the idea of new anticancer strategies based on drug repositioning of proton pumps inhibitors [67].

It is interesting to note that although SGK2 inhibition transiently blocks autophagy also in untreated and Taxol-treated cells, this block did not result in toxic effects for normal or cancer cells, therefore supporting a specific role for SGK2 inhibition in the response to PT-induced death. However, we would like to point out that SGK2, like the other members of its family, could be directly involved in the regulation of other cellular pathways like cell proliferation and apoptosis that we did not investigate here and that could also contribute to its regulation of PT-sensitivity.

Overall, we report here a new role of SGK2 kinase in the control of PT-induced cell death in different types of cancer by the inhibition of autophagy. This role seems not be shared by the two other members of the SGK family in EOC cells, although more studies, likely involving the use of CRISPR technology to knock out the different members of the family, are necessary to definitely confirm this observation. Being SGK2 a druggable kinase, amplified in a subset of EOC, for which inhibitors are already available our findings could have also an immediate translational relevance to improve the treatment and/or the quality of life of patients with PT-treatable, SGK2-expressing cancers.

## Materials and methods

### Study approval

Our institutional Biobank has collected samples and obtained informed consent from all patients. The Internal Review Board approved this study (#IRB-06/2011 and IRB-05-2014).

### Cell culture

MDAH-2774, SKOV3, TOV112D, TOV21G, OV90, MDAH-MB-468, BT-549, FaDu, CAL27 and 293T17 cells were purchased from ATCC; OVCAR8 from NCI; Immortalized Human Ovarian Epithelial cells (HuNoEOC) from ABM; COV318 from ECACC; 293FT from Invitrogen. Fresh tumor specimens were used to generate primary EOC cultures.

### Reagents

Carboplatin (CBDCA), Cisplatin (CDDP), and Taxol (TEVA Italia) were used for in vitro experiments. GSK650394 is a SGK1/SGK2 kinase inhibitor purchased from Tocris Bioscience (3572). Bafilomycin A and Acridine Orange were purchased from Sigma-Aldrich Co and L-leucyl-L-leucine methyl ester (LLOMe) was purchased from Santa Cruz (Table S1).

### Plasmids and constructs

pDONR223 SGK2 was a gift from William Hahn & David Root (Addgene plasmid #23378) and it was used to generate the pEGFP SGK2 vector, using pEGFP-C1 vector (Clontech), and pLPC SGK2 vector, using the pLPC vector gently provided by Dr. R. Maestro (CRO Aviano). Site-directed mutagenesis was used to generate SGK2 DN (SGK2 T193A/S356A) mutant, SGK2 CA (SGK2 S356D) mutant and SGK2 Kinase Dead (KD) (SGK2 K64M) (primers listed in Table S1) with QuikChange Site-Directed Mutagenesis Kit (Agilent). mRFP-EGFP-LC3 was a gift from Tamotsu Yoshimori (Addgene plasmid # 21074). pLKO shRNAs were purchased from Sigma-Aldrich Co and they are listed in Table S1.

### Cell viability assays

Cells were seeded in 96-well culture plates and, after 72 h from transduction or 24 h from inhibitor treatment, cells were treated with normal growth medium containing vehicle or CBDCA/CDDP for 16 h at the indicated concentrations. Cell viability was evaluated 24 h after the end of treatment using CellTiter 96 AQueous cell proliferation assay kit (Promega). These assays were performed three times with three biological replicates for each experimental condition. Data is depicted in some experiments as Abs value at 492 nm, in others as percentage of viable cells respect to untreated cells (set as 100% as reference) as described in figure legends. In all cases, data represent the mean ± SD.

### Preparation of cell lysates, immunoblotting, and immunoprecipitation

Cell lysates were prepared using cold RIPA lysis buffer (150 mM NaCl, 50 mM Tris HCl [ph8], 1% Igepal, 0,5% sodium deoxycholate, 0,1% SDS) plus a protease inhibitor cocktail (Complete, Roche), 1 mM sodium orthovanadate, and 1 mM dithiothreitol as previously reported [68]. Protein concentrations were determined using the Bio-Rad protein assay (Bio-Rad). For immunoblotting, equal concentrations of protein samples (60 μg) were separated by 4–20% SDS-PAGE (Criterion precast gel; Bio- Rad) and transferred to nitrocellulose (Hybond C; Amersham) or PVDF (Bio-Rad) membranes. Immunoprecipitations were performed using 1 mg of cell lysate in HNTG buffer (20 mM HEPES, 150 mM NaCl, 10% glycerol, 0.1% Triton X-100) supplemented with protease inhibitors plus 2 μg of the indicated specific primary antibody and incubating overnight at 4 °C. The immunocomplexes were captured by incubation with protein A and G beads (GE Healthcare) for 2 h at 4 °C, washed with HNTG buffer, eluted in 3X sample buffer, and separated on SDS-PAGE for western blot analysis. Immunoblotting was performed using the following primary antibodies: rabbit monoclonal anti-SGK2 (1:500), rabbit monoclonal anti-SGK3 (1:500), rabbit monoclonal LC3B (1:500), rabbit polyclonal anti-Caspase 9 (1:500) from Cell Signaling Technology and rabbit polyclonal LC3B (1:10000) from Novus Biological; mouse monoclonal anti-Vinculin (1:1000), mouse monoclonal anti-LAMP2 (1:200), mouse monoclonal anti-SGK1 (1:200), mouse monoclonal anti-V-ATPase H (ATP6V1H) (1:200), purchased from Santa Cruz Biotechnology; mouse polyclonal anti-SGK2 (used for IP) and mouse monoclonal anti-Cathepsin D (1:500) from Sigma Aldrich Co; rabbit monoclonal anti-p62 (1:20000) and rabbit polyclonal anti-TCIRG1/ATP6V0A3 (1:500) from Abcam; rabbit polyclonal anti-Cathepsin L (1:500) and rabbit polyclonal anti-Cathepsin B (1:500) from Elabscience; mouse monoclonal anti-GST (1.500) from BD Biosciences; mouse anti-GRB2 (1:500) from Transduction Lab; mouse monoclonal anti-GFP (1:500) from Roche. Antibodies were visualized with appropriate horseradish peroxidase-conjugated secondary antibodies (GE Healthcare) for ECL detection (Bio-Rad) or Alexa-conjugated secondary antibodies (Invitrogen) for Odyssey infrared detection (LI-COR Biosciences).

### In vitro kinase assay

In vitro kinase assays were performed by incubating SGK2 active kinase (Signal Chem) with endogenous ATP6V1H immunoprecipitated from untreated and CBDCA-treated MDAH cells (CBDCA 500 μg/ml, 16 h) or Rb1-GST recombinant protein, as substrates, for 30 min at 30 °C in the presence of 1.5 μCi of γ^32^P-ATP (PerkinElmer), as previously described [69].

### qRT–PCR analyses

qRT–PCR analyses were performed as previously described [68]. Briefly, total RNA was extracted using Trizol reagent (Invitrogen), quantified using NanoDrop (Thermo Fisher Scientific Inc., USA), and retro-transcribed using the AMV reverse transcriptase (Promega). cDNAs were amplified using SsoFast EvaGreen Supermix (Biorad) and CFX96^TM^ Real-Time PCR Detection System (Bio-Rad). SGK2 expression was normalized using SGK2/housekeeping gene as SQ mean ratio. The SQ (Starting Quantity) was calculated using CFX96^TM^ software (Bio-Rad).

### Immunofluorescence

For immunofluorescence, cells plated on coverslips and fixed in PBS 4% paraformaldehyde (PFA) were stained with primary antibodies, such as SGK2 (1:400) and LAMP2 (1:200) (Santa Cruz Biotechnologies), LC3B (1:200) (Cell Signaling), LGALS1 (1:1000) (Abcam) and p62 (1:200) (Abcam). Propidium iodide (5 μg/ml) was used for nuclear staining as reported [38, 70]. For acridine orange staining, cells were incubated with acridine orange solution (Sigma-Aldrich Co) for 30 min at 37 °C before image acquisition. Cells were treated with Bafilomycin A 0.2 µM for 30 min at 37 °C for controls. Stained cells were analyzed using a confocal laser-scanning microscope (TCS SP8 Leica). Fluorescence intensity and protein co-localization were studied using the Volocity® software (PerkinElmer) (See also Supplementary Materials and methods).

### Statistical analyses

Statistical significance (*p* < 0.05), means, standard deviation, 95% confidence intervals (CIs) were determined by using GraphPad PRISM software (version 6.0) using the most appropriate test, as specified in each figure. Statistical significance was indicated with: *p < 0.05, **p < 0.01, ***p < 0.001, ****p < 0.0001

SGK2 expression in available ovarian cancer dataset is reported from Oncomine website (http://www.oncomine.org), statistical analysis is described in each figure [59, 60].

## Supplementary information

Supplementary Information

Supplementary Table S1

Data File S1

Figure S1

FigureS2

Figure S3

Figure S4

Figure S5

Figure S6

Figure S7

Figure S8

Figure S9

Figure S10

Figure S11
