# Supplementary material for: Serum- and glucocorticoid- inducible kinase 2, SGK2, is a novel autophagy regulator and modulates platinum drugs response in cancer cells
Source: Oncogene. 2020 Aug 27;39(40):6370–86. doi: 10.1038/s41388-020-01433-6 (PMC7529585; doi:10.1038/s41388-020-01433-6)
Supplement: Supplementary file 1 — Supplementary Information [file 41388_2020_1433_MOESM1_ESM.docx]

**Serum- and Glucocorticoid- inducible Kinase 2, SGK2, is a novel autophagy factor and modulates platinum drugs response in cancer cells.**

Valentina Ranzuglia, Ilaria Lorenzon, Ilenia Pellarin, Maura Sonego, Alessandra Dall’Acqua, Sara D’Andrea, Sara Lovisa, Ilenia Segatto, Michela Coan, Jerry Polesel, Diego Serraino, Patrizia Sabatelli, Paola Spessotto, Barbara Belletti, Gustavo Baldassarre and Monica Schiappacassi.

**Supplementary Information.**

**Supplementary Data File S1:** Loss-of-function screening: library targets, raw data and analyzed data. Validation screening results (File Excel composed of 9 different sheets/Sections preceded by a “Read Me” Section for guiding the analysis of the Data File)

**Supplementary Materials and methods** (File Word)

**Supplementary Table S1**: Reagents and resources utilized in this work (File Word)

**Supplementary Figures:**

**Fig. S1** (related to Fig 1). SGK2 silencing sensitizes EOC cells to platinum treatment.

**Fig. S2** (related to Fig 1). SGK1 and SGK3 silencing do not sensitize EOC cells to platinum treatment.

**Fig. S3** (related to Fig 2). SGK2 silencing sensitizes EOC cells via a kinase –dependent mechanism.

**Fig.** **S4** (related to Fig 3). SGK2 silencing sensitizes EOC cells via a kinase –dependent mechanism: Patients data table.

**Fig. S5** (related to Fig 4). SGK2 inhibition induces the accumulation of autophagic vesicles.

**Fig. S6** (related to Fig 4). TEM analysis at low magnification, SGK2 inhibition alters autophagic flux in TNBC and HNSCC cells.

**Fig. S7** (related to Fig. 6). SGK2 is localized in autophagolysosomes.

**Fig. S8** (related to Fig 7). SGK2 inhibition causes autophagy blockade altering activation of lysosomal cathepsins. SGK2 binds and phosphorylates the V-ATPase proton pump V1H subunit.

**Fig. S9** (related to Fig 8). CBDCA treatment induces autophagy stimulation

**Fig S10** (related to Fig 8) CBDCA treatment induces autophagy stimulation.

**Fig. S11** (related to Fig 8) SGK2 in available gene expression dataset.

**Supplementary Figure Legends**

**Fig. S1** (related to Fig. 1). SGK2 silencing sensitizes EOC cells to platinum treatment. **a** Schematic representation of the functional genomic and validation screenings performed in EOC cell lines that resulted in the identification of SGK2 silencing as platinum sensitizer. **b** Graph reports the viability of transduced cells with control (Ctrl) and SGK2 shRNAs in the validation screening performed using the indicated EOC cell lines. Cell viability is expressed as the ratio between treated/untreated and represents the mean ± SD of three biological replicates. **c,d,e** Viability of TOV21G (**c**), OV90 (**d**) and SKOV3 (**e**) cells transduced with control (Ctrl) and SGK2 shRNAs, untreated or treated respectively with CBDCA 100, 80 and 150 μg/ml for 16 hours. SGK2 silencing was confirmed by Western Blot (WB) (lower panels). Vinculin was used as loading control. In the figure data are expressed as mean ± SD of three biological replicates. Significance was calculated using unpaired, two-tailed Student t-test comparing silenced cells vs the corresponding parental cell line. ***p<0.001, **p<0.01. *p<0.05.

**Fig. S2** (related to Fig. 1). SGK1 and SGK3 silencing do not sensitize EOC cells to platinum treatment. **a**. Nonlinear regression analyses of cell viability assay in SGK2 silenced MDAH and TOV21G cells treated with increasing doses of CDBCA for 16 h. Results are expressed as percentage of viable cells respect to the untreated cells and represent the mean (±SD) of 3 biological replicates. The tables report the IC50s and the confidence interval (C.I.) for each condition. Fisher's exact test was used to calculate the global *p* value reported in the graphs. **b,c** Graph reports the viability of MDAH cells transduced with control (Ctrl) and SGK3 shRNAs (**b**) or with control (Ctrl) and SGK1 shRNAs (**c**), and treated or not with CBDCA 140 µg/ml for 16 hours. Immunoblot showing SGK3, SGK2 and SGK1 expression levels are reported (right panels). Vinculin was used as loading control.

**Fig. S3** (related to Fig. 2). SGK2 silencing sensitizes EOC cells via a kinase –dependent mechanism. **a** Schematic representation of SGK2 kinase dead (KD) construct (pLPC-SGK2^K64M^) (left, upper panel). In the lower western blot, the expression of SGK2 KD protein in TOV21G cells transfected as indicated (e.v. = empty vector). Vinculin was used as loading control. The graph on the right reports cell viability of TOV21G cells transfected with e.v. or SGK2 KD and then treated for 16 hours with CBDCA as indicated. Results are expressed as survival ratio (%) between CBDCA treated and untreated cells (set as 100% as reference). **b** Graph reports cell viability of MDAH treated for 24 hours with different doses of GSK650394 (GSK), and in combination with cisplatin (CDDP) 3 μg/ml for 16 hours. Experimental timeline is shown (right panel). **c** Nonlinear regression analyses of cell viability assays of MDAH and TOV21G cells treated for 24 hours with different doses of GSK650394 (GSK) and combined with increasing doses of CBDCA for 16 hours. Results are expressed as percentage of viable cells respect to the untreated cells and represent the mean (±SD) of 3 biological replicates. The tables report the IC50s and the confidence interval (C.I.) for each condition. Fisher's exact test was used to calculate the global *p* value reported in the graphs. **d** Graph reports the cell viability of COV318 cells treated with different doses of GSK650394 (GSK) for 36 hours and combined with increasing doses of CBDCA for 16 hours **e** Graph reports cell viability of TOV21G cells transfected with SGK2 DN or the empty vector (e.v.) and then treated with GSK650394 35 µM (GSK) (36h), CBDCA 100 µg/ml (16h) and GSK+CBDCA. In the figure data are mean ± SD of three biological replicates. Significance was calculated using unpaired, two-tailed Student t-test. ****p<0.0001, ***p<0.001, **p<0.01; ns, not significant.

**Fig.** **S4** (related to Fig. 3). SGK2 silencing sensitizes EOC cells via a kinase –dependent mechanism: Patients data table. Histo-pathological and clinical data of ovarian cancer patients. Table reports grade, histotype and chemotherapy (when applied) of samples analyzed in Fig. 3.

**Fig. S5** (related to Fig. 4). SGK2 inhibition induces the accumulation of autophagic vesicles. **a** Experimental timeline design for optical microscopy studies (OM), in MDAH and TOV21G cells treated with GSK650394 35 µM (GSK), CBDCA (140 µg/ml and 100 µg/ml, respectively) and GSK+CBDCA. **b** Typical images from optical microscopy studies of MDAH, TOV21G and TOV112D cells treated as reported in **a** and indicated in each panel. Scale bars=50 µm. **c** Typical images from optical microscopy studies of MDAH and TOV21G cells untreated, treated with GSK650394 35 µM (GSK) for 36 hours, and then released in complete growth medium (24 hours after GSK removal), as indicated. Scale bars=50 µm. **d** Typical images from optical microscopy studies of MDAH and TOV21G cells transduced with control (Ctrl) or SGK2 shRNAs (upper panels). SGK2 expression in transduced cells is reported in lower WB. Vinculin was used as loading control. Black arrows point to intracellular vesicles present in SGK2 silenced cells. Scale bars=50 µm. **e** Representative optical microscopy pictures of MDA-MB-468, BT-549 (upper panels), FaDu and CAL27 (lower panels) cells treated or not with GSK650394 35 μM for 36 hours. Cytoplasmic vesicles were observed in GSK-treated cells. Scale bars=50 µm. **f** Quantification of vacuolized cells after GSK650394 treatment. Results are expressed as % of vacuolized cells/field (mean ± SD, 5 fields/condition were analyzed). n.m. indicates non measurable (less than 2 vacuolized cells /field).

**Fig. S6** (related to Fig. 4). TEM analysis at low magnification, SGK2 inhibition alters autophagic flux in TNBC and HNSCC cells. **a** TEM ultrastructure analysis at low magnification of MDAH (left) and TOV21G (right) cells. Scale bar: 2.5 μ. **b** Graph reporting cell viability of MDAH cells treated with Bafilomycin A1 (Baf A1) and CBDCA 140 µg/ml as depicted in the experimental timeline above the graph. **c** Western Blot analysis of the autophagy markers LC3I/LC3II and p62 in MDAH cells, treated with GSK650394 35 μM (GSK), CBDCA 140 μg/ml or CDDP 3 μM and respective combination treatment. Vinculin was used as loading control. **d** WB analyses evaluating the expression of the autophagy markers LC3I/LC3II and p62 in MDA-MB-468, BT-549, FaDu and CAL27 cells, treated with GSK650394 35μM (36 hours), CBDCA (16 hours) and GSK+CBDCA as in Fig 3d. Vinculin was used as loading control.

**Fig S7** (related to Fig. 6). SGK2 is localized in autophagolysosomes. Representative immunofluorescence staining evaluating the expression and localization of LAMP2 (**a**) or SGK2 (**b**) (green) and p62 (red) in MDAH cells treated or not with GSK650394 35 μM for 36 hours (GSK). Nuclei were stained with propidium iodide and pseudo colored in blue. Scale bars: 10 μm. In (**a-b**) right panels indicates ROIs (white dotted lines) chosen for co-localization analysis, for each ROI the corresponding value of Pearson correlation is reported on right tables. xzy projections are also displayed. Scale white bars: 10 μm.

**Fig. S8** (related to Fig. 7). SGK2 inhibition causes autophagy blockade altering activation of lysosomal cathepsins. SGK2 binds and phosphorylates the V-ATPase proton pump V1H subunit. **a** WB analyses of Cathepsin L and B (CTSL and CTSB) expression in MDAH treated with GSK650394 35 μM (GSK) for the indicated time points. Vinculin was used as loading control. **b** WB analyses of p62, Cathepsin D (CTSD), LC3I/LC3II and SGK2 in OVCAR8 stably overexpressing the empty vector (e.v.) or SGK2. Vinculin was used as loading control. **c** Table reporting the *in-silico* analysis of the Serine/Threonine residues of ATP6V1H and ATP6V0A3 subunits of V-ATPase predicted to be phosphorylated by SGK2 using the PhosphoNET kinase predictor scores. A score >100 indicates that a phosphorylation site could be a reasonable target for the kinase of interest. **d** Immunoprecipitation (IP) analysis of endogenous SGK2 in MDAH cells treated or not with GSK650394 35 μM for 16 hours (GSK). IPs and corresponding lysates (INPUT) were evaluated by WB for the expression of ATP6V1H and SGK2. IgG indicates IP with unrelated antibody. Vinculin was used as loading control. The amount of ATP6V1H co-immunoprecipitated with SGK2, obtained by densitometric analyses, is expressed as fold respect to untreated cells and is reported in the right graph. **e** *In vitro* kinase assay performed using active SGK2-GST and Rb-GST recombinant proteins. Reaction mix control is present in the first line (right panel). WB analysis reporting the expression of the used SGK2-GST and Rb-GST recombinant proteins is shown in the left panel.

**Fig. S9** (related to Fig 8). CBDCA treatment induces autophagy stimulation. **a** WB analyses reporting the expression of p62 in MDA-MB-468, BT-549, FaDu and CAL27 cells treated with CBDCA as indicated. **b** Combined treatment GSK650394+CBDCA renders MDAH cells prone to apoptotic cell death. WB analysis of p62, LC3I/II, caspase 9 and PARP1 in MDAH cells treated as indicated and analyzed after treatment and after 24h & 36h of release. Experiment time line is shown. Vinculin (**a,b,**) was used as loading control.

**Fig S10** (related to Fig 8). CBDCA treatment induces autophagy stimulation. **a,b** WB analyses of p62 and LC3I/II expression in MDAH cells treated with increasing doses of CBDCA (**a**) and taxol (**b**) for 16-24 and 36 h, with and without GSK combined treatment. Vinculin (**a,b,**) was used as loading control. **c** Nonlinear regression analyses of cell viability assay in MDAH cells treated with GSK650394 (GSK) for 36 hours and combined with increasing doses of Taxol for 16 hours. Results are expressed as percentage of viable cells respect to the un treated cells and represent the mean (±SD) of 3 biological replicates. The table reports the IC50s and the confidence intervals (C.I.) of each condition. Fisher’s exact test was used to calculate the global *p* value reported in the graph.

**Fig. S11** (related to Fig. 8) SGK2 data in available ovarian cancer gene expression dataset. Oncomine database was used to explore SGK2 expression (**a,b)** and amplification (**c**) vs normal tissue, number of samples are indicated between parentheses.

**Supplementary Materials and methods**

*Cell culture*

Ovarian Cancer cell lines and primary cultures were maintained in RPMI-1640 medium. HuNoEOC cells were grown in Prigrow I medium. FaDu and CAL27 (HNSCC cells), MDA-MB-468 and BT-549 (TNBC cells), 293FT cells (used for lentivirus production) were grown in DMEM. All media were supplemented with 10% heat-inactivated FBS (Sigma-Aldrich Co). Mycoplasma contamination was assessed every 15 days using the MycoAlert test (Lonza). Cell lines were authenticated according to the Cell ID TM System (Promega) protocol and using Genemapper ID Ver 3.2.1. Last authentication was performed in 2018. Cells were kept frozen, and used within 2 months of culture to perform the experiments.

*Loss of function screening*

Loss-of-function screening was performed using a CBDCA dose able to cause a 10-20% of cell mortality previously determined for each cell line after 16 h treatment and evaluating cell viability 24 hours later. Briefly: on day 1, 1000 MDAH cells/well and 700 SKOV3 cells/well were seeded in 96-well plates; on day 2, cells were transduced in duplicate with three shRNAs for each of the chosen 680 genes (2040 shRNAs); 72 hours post transduction, one 96-well plate was treated with CBDCA (140μg/ml for MDAH and 150µg/ml for SKOV3) for 16 hours and the second one treated with vehicle as control. Cell viability was evaluated 24 hours after the end of treatment using CellTiter 96 AQueous kit (Promega). The screening was performed twice on each cell line and the statistical analyses were used to identify significant hits. List of target genes, raw and analyzed data of the loss of function screening performed on MDAH and SKOV3 cells are reported in Data File S1 (Excel composed of 9 different Sections/sheets). We chose 3 different commercial libraries from Mission Sigma targeting the pathways involved in platinum cell response such as: apoptosis, Human DNA repair and Human p53 pathway (target genes are listed in Section 1, Data File S1). Complete library was delivered by Sigma in 22 96wells plates (Section 2). Raw data and statistical analysis of the loss of function screening performed are reported in Sections 3&5 for MDAH cells and Sections 6&7 for SKOV3 cells in Data File S1. Each round of screening was run together with a plate containing negative and positive controls and these results were used to determine the quality of our screenings. These positive and negative controls were chosen according our previous experience. As controls we included molecules that we have already described as presenting (or not) synthetic lethality with platinum drugs when silenced (e.g. positive controls: p53, stathmin ^1^ CDK6 and FOXO3a, ^2^). As example, quality control parameters for MDAH screening are included in the Section 5. Strictly Standardized Mean Difference (SSMD) was calculated on log2 (untreated/treated) for positive and negative controls: this value was always higher than 1.7 indicating appropriate quality of the screenings performed ^3^. Pre-specified condition established to consider as positive hits those genes for which at least two shRNAs were able to increase platinum-induced cell death >2 folds respect to control shRNA. The results of the first screening were then validated in a second screening performed using five shRNAs for each gene in four different EOC cell lines: MDAH, SKOV3, TOV112D and OV90 cell lines. We took into account only the genes for which at least three out of five shRNAs displayed a significantly enhanced survival after platinum treatment respect to controls in at least three different cell lines. List of hits unveiled after primary screenings is presented in Data File S1, Section 8 “Validation screening targets”, and Validation screening results are depicted in Section 9.

To identify shRNAs that are synthetically lethal with CBDCA treatment log2 signal of the non-treated samples were compared to that of the treated to derive the log2(non-treated/treated) ratios for each well in the twenty-two 96-well-plate among the two screening replicates. This ratio represents the changes in an shRNA’s relative abundance between the non-treated and treated samples, with positive value indicating synthetic lethality effect and negative value a protective effect against CBDCA treatment.

We applied the Z-score normalization to account for plate-to-plate variation ^4^. Statistical analysis in order to correctly select the candidate shRNA were conducted using a custom statistical package based on the Linear Models for Microarray data (Limma) method. shRNAs that presented synthetic lethality or protective effect were ranked using a moderated t test statistic ^5^. To account for multiple testing problem Benjamini and Hochberg's method ^6^ was applied to control the false discovery rate at a level of 5%. shRNAs that yielded synthetic lethality were filtered to identify genes that were targeted by multiple shRNAs.

*Transfections, lentiviral preparation, transduction methods*

OVCAR8 cells were transfected with pEGFP or pEGFP SGK2 vectors and TOV21G cells with pLPC empty vector, pLPC-SGK2 KD, pLPC SGK2 DN, pLPC SGK2 CA and pLPC SGK2 wild type using FuGENE HD Transfection Reagent (Promega). OVCAR8 pEGFP or pEGFP SGK2 cells were then selected in the presence of G418 (Sigma-Aldrich Co) 0.5 mg/ml to obtain a stably overexpressing cell population. TOV21G cells were then selected in the presence of puromycin (Sigma-Aldrich Co) 1mg/ml to obtain a stably overexpressing cell population. MDAH cells were transfected with mRFP-EGFP-LC3 vector using Lipofectamine 2000 (Invitrogen).

To produce lentiviral particles, 293FT cells were transfected using calcium phosphate method using with the lentiviral-based shRNA constructs and lentiviral system vectors pLP1, pLP2, and pVSV-G (Invitrogen) as described ^2^. The lentiviral particles were collected from the culture medium of these cells after 48 hours and 72 hours from transfection to transduce cells, supplementing the medium with 8 mg/ml polybrene (Sigma-Aldrich Co). Viral particles-containing medium was removed after 16 hours. Transduced cells were lysed 72 hours after transduction and the knockdown efficiency was confirmed by immunoblot analysis.

*Transmission electron microscopy*

MDAH and TOV21G were plated on coverslips, treated with GSK650394 35µM for 36 hours, and fixed in 2,5% glutaraldehyde in 0.1M cacodylate buffer pH 7.4. Post-fixation was conducted in 1% osmium tetroxide and 1,6% potassium ferricyanide for 2h at 4°C. The samples were dehydrated in alcohol and included in resin (Epon812). Ultrafine sections were obtained cutting the monolayers en face. The samples were stained with uranyl acetate and Reynold’s lead citrate, and analyzed with a transmission electron microscope (TEM, PhilipsEM400 at 100kV). >50 cells were evaluated for each experimental condition.

*Acridine Orange Staining & Immunofluorescence*

For acridine orange staining, cells were incubated with acridine orange solution (1 μg/ml, Sigma-Aldrich Co, A8097) in DMEM without red phenol for 30 minutes at 37° C before microscope acquisition. Cells were treated with Bafilomycin A 0.2 µM for 30 minutes at 37° C as control.

Images were acquired with a true confocal scanner system (TCS SP8 FSU AOBS, Leica Microsystems), equipped with a Leica DMi8 inverted microscope (Leica Microsystems) and a box live imaging system with an incubator box combined with a precision air heater for a tightly controlled temperature (37°C) and a device for CO_2_ and humidity control (Life Imaging Services, Basel, Switzerland). For acridine orange visualization a HC PL APO CS2 63x/1.40 oil objective a 488 laser line (5% power intensity) and two standard detectors (PMT1 490-540 nm, gain 477; PMT2 549-675 nm, gain 445) were used. The analysis was performed on single slices obtained with the Leica confocal LAS AF SP8 software and corresponding to the best focus plane of the sample. For each sample at least ten different fields containing about 25-30 cells were acquired. The red-to green fluorescence intensity ratio (R/GFIR) associated to the whole cells (according to Thomè et al 2016) was calculated for each acquired filed by Volocity software (Perkin Elmer) and the media ± SD were reported in Fig 5b.

*In silico prediction of phosphorylation sites*

Prediction of phosphorylation sites of SGK2 on ATP6V1H and ATP6V0A3 subunits of V-ATPase were obtained using the PhosphoNET algorithm (Kinexus Bioinformatics Corporation). PhosphoNET is a repository of known and predicted information on human phosphorylation sites and the identities of protein kinases that may target these sites. PhosphoNET kinase substrate predictions are based on scoring matrices assigned to amino acid frequency in the consensus phosphorylation site of protein kinases. The specificity matrices are generated directly from the primary amino acid sequences of the catalytic domains of these kinases. The higher the indicated score for a putative phosphorylation site, the better the prospect that a kinase could phosphorylate that given site of the substrate protein. A PhosphoNET prediction score >100 indicates that a phosphorylation site could be a reasonable target for the kinase of interest.

1 Sonego M, Schiappacassi M, Lovisa S, Dall’Acqua A, Bagnoli M, Lovat F *et al.* Stathmin regulates mutant p53 stability and transcriptional activity in ovarian cancer. *EMBO Mol Med* 2013; **5**: 707–722.

2 Dall’Acqua A, Sonego M, Pellizzari I, Pellarin I, Canzonieri V, D’Andrea S *et al.* CDK6 protects epithelial ovarian cancer from platinum-induced death via FOXO3 regulation. *EMBO Mol Med* 2017; **9**: 1415–1433.

3 Zhang XD. A pair of new statistical parameters for quality control in RNA interference high-throughput screening assays. *Genomics* 2007; **89**: 552–561.

4 Malo N, Hanley JA, Cerquozzi S, Pelletier J, Nadon R. Statistical practice in high-throughput screening data analysis. *Nat Biotechnol* 2006; **24**: 167–175.

5 Smyth GK. Linear models and empirical bayes methods for assessing differential expression in microarray experiments. *Stat Appl Genet Mol Biol* 2004; **3**: Article3.

6 Benjamini Y, Hochberg Y. Controlling the false discovery rate: a practical and powerful approach to multiple testing. *J R Stat Soc Ser B Methodol* 1995; : 289–300.
