# Supplementary material for: Serum- and glucocorticoid- inducible kinase 2, SGK2, is a novel autophagy regulator and modulates platinum drugs response in cancer cells
Source: Oncogene. 2020 Aug 27;39(40):6370–86. doi: 10.1038/s41388-020-01433-6 (PMC7529585; doi:10.1038/s41388-020-01433-6)
Supplement: Supplementary file 2 — Supplementary Table S1 [file 41388_2020_1433_MOESM2_ESM.docx]

| **REAGENT or RESOURCE** | | **SOURCE** | | **IDENTIFIER** |
| --- | --- | --- | --- | --- |
| **Antibodies** | | | | |
| Rabbit monoclonal anti-SGK2 (D7G1) | | Cell Signaling Technology | | 7499S |
| Mouse polyclonal anti-SGK2 (3D2) | | Sigma-Aldrich | | SAB1408718 |
| Mouse monoclonal anti-SGK1 (G-4) | | Santa Cruz Biotechnology | | sc-377360 |
| Rabbit monoclonal anti-SGK3 (D42C2) | | Cell Signaling Technology | | 8573S |
| Rabbit monoclonal anti-SQSTM1/p62 (EPR4844) | | Abcam | | ab109012 |
| Rabbit monoclonal anti-LC3B (D11) | | Cell Signaling Technology | | 3868 |
| Mouse monoclonal anti-LAMP2 (H4B4) | | Santa Cruz Biotechnology | | sc-18822 |
| Mouse monoclonal anti-Cathepsin D (CTD-19) | | Sigma-Aldrich | | C0715 |
| Rabbit polyclonal anti-Caspase 9 | | Cell Signaling Technology | | 9502S |
| Rabbit polyclonal anti-Cathepsin B | | Elabscience | | E-AB-30772 |
| Rabbit polyclonal anti-Cathepsin L | | Elabscience | | E-AB-33394 |
| Mouse monoclonal anti-V-ATPase H (C-8) | | Santa Cruz Biotechnology | | sc-20950 |
| Rabbit polyclonal anti-TCIRG1/ATP6V0A3 | | Abcam | | ab139812 |
| Mouse monoclonal anti-GFP | | Roche | | 11814460001 |
| Mouse monoclonal anti-GST (G172-1138) | | BD Biosciences | | 554805 |
| Mouse monoclonal anti-GRB2 | | Transduction Lab | | 610111 |
| Rabbit polyclonal anti-LGALS1 | | Abcam | | ab25138 |
| Rabbit polyclonal anti-LC3B | | Novus Biologicals | | NB100-2220 |
| Mouse monoclonal anti-Vinculin (7F9) | | Santa Cruz Biotechnology | | sc-73614 |
| Horseradish peroxidase-conjugated secondary antibodies | | GE Healthcare | | NA934V  NA931V |
| Alexa-conjugated secondary antibodies | | Invitrogen | | A-21109  A-32729 |
| **Bacterial and Virus Strains** | | | | |
| E. coli DH5α | | ThermoFisher Scientific | |  |
| **Chemicals, Peptides, and Recombinant Proteins** | | | | |
| Carboplatin (CBDCA) | | TEVA Italia |  | |
| Cisplatin (CDDP) | | TEVA Italia |  | |
| GSK650394 | | Tocris Bioscience | 3572; CAS:890842-28-1 | |
| Bafilomycin A1 | | Sigma-Aldrich | B1793 | |
| Taxol® Paclitaxel | | Actavis, Ireland |  | |
| Polybrene | | Sigma-Aldrich | H9268 | |
| Puromycin dihydrochloride | | Sigma-Aldrich | P9620 | |
| G418 disulfate salt | | Sigma-Aldrich | A1720 | |
| nProteinA Sepharose 4 Fast Flow | | GE Healthcare | 17-5280-01 | |
| Protein G Sepharose 4 Fast Flow | | GE Healthcare | 17-0618-01 | |
| Active SGK2 recombinant protein | | Signal Chem | S07-10G | |
| γ^32^P‐ATP | | PerkinElmer | NEG002A | |
| Lipofectamine 2000 | | Invitrogen | 11668019 | |
| Lipofectamine 3000 | | Invitrogen | L3000015 | |
| L-leucyl-L-leucine methyl ester (LLOMe) | | Santa Cruz | sc-285992 | |
| Acridine orange solution | | Sigma-Aldrich | A8097 | |
| **Critical Commercial Assays** | | | | |
| CellTiter96 AQueous cell proliferation assay (MTS) | Promega | | | G3582 |
| Bio-Rad protein assay | Bio-Rad | | | 500-0006 |
| **Experimental Models: Cell Lines** | | | | |
| Human: MDAH2774 cells | | ATCC | | CRL-10303 |
| Human: TOV21G cells | | ATCC | | CRL-11730 |
| Human: SKOV3 cells | | ATCC | | HTB-77 |
| Human: OVCAR8 cells | | NCI | | CVCL_1629 |
| Human: COV318 | | ECACC | | 07071903 |
| Human: TOV112D | | ATCC | | CRL-11731 |
| Human: OV90 | | ATCC | | CRL-11732 |
| Human: Immortalized Human Ovarian Epithelial cells (HuNoEOC) | | ABM | | T1074 |
| Human: MDA-MB-468 | | ATCC | | HTB-132 |
| Human: BT-549 | | ATCC | | HTB-122 |
| Human: FaDu | | ATCC | | HTB-43 |
| Human: CAL27 | | ATCC | | CRL-2095 |
| Human: 293FT | | Invitrogen | | R700-07 |
| **Oligonucleotides** | | | | |
| SGK2 T193A:  Forward  5’ GAGCCTGAAGACACCACATCCGCATTCTGTGGTACCCCTGAGTAC 3’  Reverse  5’ GTACTCAGGGGTACCACAGAATGCGGATGTGGTGTCTTCAGGCTC 3’ | | This paper | | N/A |
| SGK2 S356A:  Forward  5’ CAAGTGCATTCCTGGGATTTGCTTATGCGCCAGAGGATGATGAC 3’  Reverse  5’ GTCATCATCCTCTGGCGCATAAGCAAATCCCAGGAATGCACTTG 3’ | | This paper | | N/A |
| SGK2 K64M  Forward  5’ GGGGCGTTCTATGCAGTGATGGTACTACAGAAAAAGTCC 3’  Reverse  5’ GGACTTTTTCTGTAGTACCATCACTGCATAGAACGCCCC 3’ | | Pao et al., 2010 | | N/A |
| SGK2 CA S356D:  Forward:  5' gtgcattcctgggatttgattatgcgccagaggatgatg  Reverse:  5' catcatcctctggcgcataatcaaatcccaggaatgcac | | This paper | | N/A |
| SGK2 qRT  Forward:  5' GCTCGACTATGTCAACG 3'  Reverse:  5' CCAAGAGAATGTTCTCTGG 3' | | This paper | | N/A |
| **Recombinant DNA** | | | | |
| pDONR223 SGK2 | | Johannessen et al., 2010 | | Gift from William Hahn & David Root (Addgene plasmid #23378) |
| pLP1 | | Invitrogen | |  |
| pLP2 | | Invitrogen | |  |
| pVSV‐G | | Invitrogen | |  |
| pLKO SGK1 shRNA 8 | | Sigma-Aldrich | | SHCLNG-NM_005627  TRCN0000040175 |
| pLKO SGK1 shRNA 9 | | Sigma-Aldrich | | SHCLNG-NM_005627  TRC0000009867 |
| pLKO SGK2 shRNA 1 | | Sigma-Aldrich | | SHCLNG-NM_170693  TRCN0000272924 |
| pLKO SGK2 shRNA 2 | | Sigma-Aldrich | | SHCLNG-NM_170693  TRCN0000272861 |
| pLKO SGK2 shRNA 3 | | Sigma-Aldrich | | SHCLNG-NM_170693  TRCN0000272863 |
| pLKO SGK2 shRNA 4 | | Sigma-Aldrich | | SHCLNG-NM_170693  TRCN0000002111 |
| pLKO SGK2 shRNA 5 | | Sigma-Aldrich | | SHCLNG-NM_170693  TRCN0000002112 |
| pLKO SGK3 shRNA 1 | | Sigma-Aldrich | | SHCLNG-NM_01357  TRCN0000001517 |
| pLKO SGK3 shRNA 2 | | Sigma-Aldrich | | SHCLNG-NM_01357  TRCN0000001518 |
| pEGFP-C1 | | Clontech | | 6084-1 |
| mRFP-GFP-LC3 | | Kimura et al., 2007 | | Gift from Tamotsu Yoshimori (Addgene plasmid#21074) |
| **Software and Algorithms** | | | | |
| PRISM | | GraphPad | | N/A |
| Image Lab software | | Biorad | | www.bio-rad.com/en-no/product/image-lab-software |
| Volocity® software | | PerkinElmer | | N/A |
| PhophoNET | | Kinexus Bioinformatics Corporation | | http://www.phosphonet.ca/ |

**Table** **S1.** Reagents and other resources utilized in this work.
