# Supplementary figures and images for: Serum- and glucocorticoid- inducible kinase 2, SGK2, is a novel autophagy regulator and modulates platinum drugs response in cancer cells
Source: Oncogene. 2020 Aug 27;39(40):6370–86. doi: 10.1038/s41388-020-01433-6 (PMC7529585; doi:10.1038/s41388-020-01433-6)

**Fig. S1 (related to Fig. 1)****a**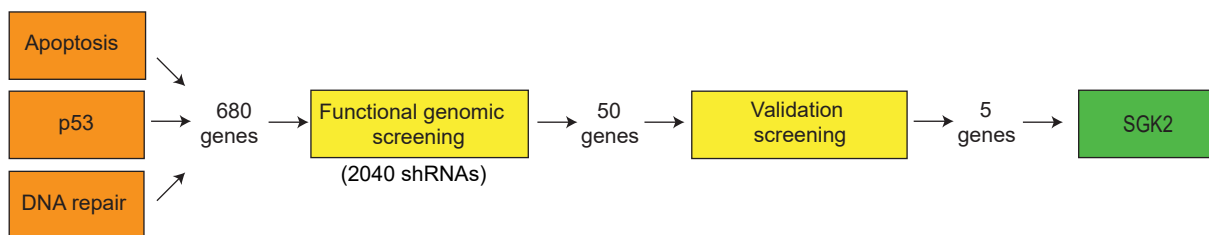**b**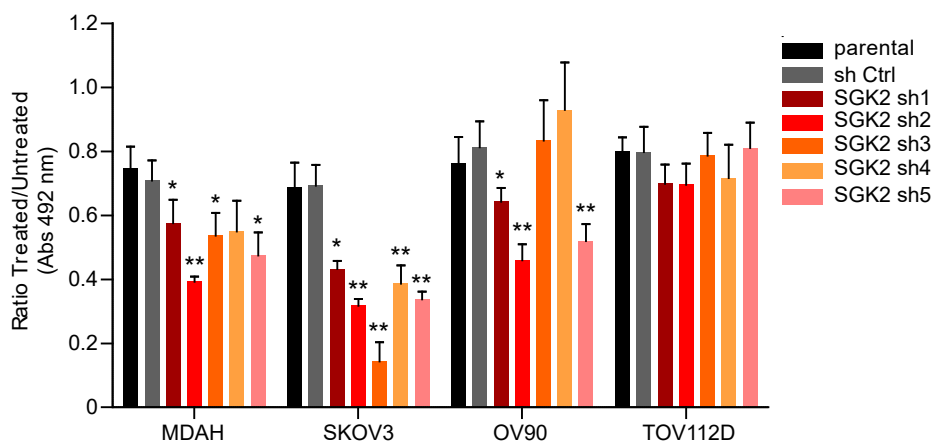**c**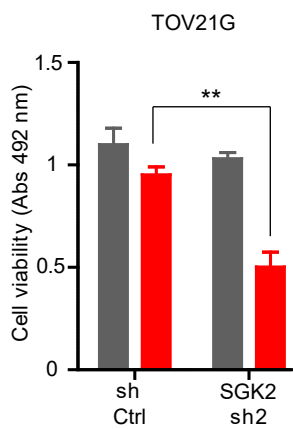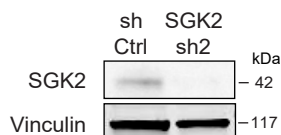**d**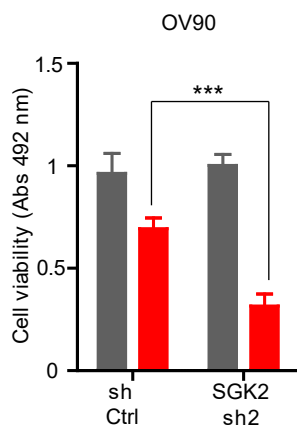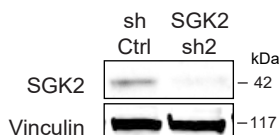**e**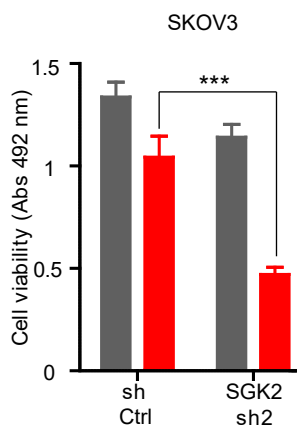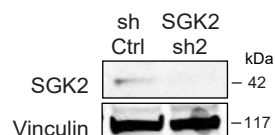

Supplement: Supplementary file 4 — Figure S1 [file 41388_2020_1433_MOESM4_ESM.pdf]

**Fig. S2 (related to Fig. 1)**

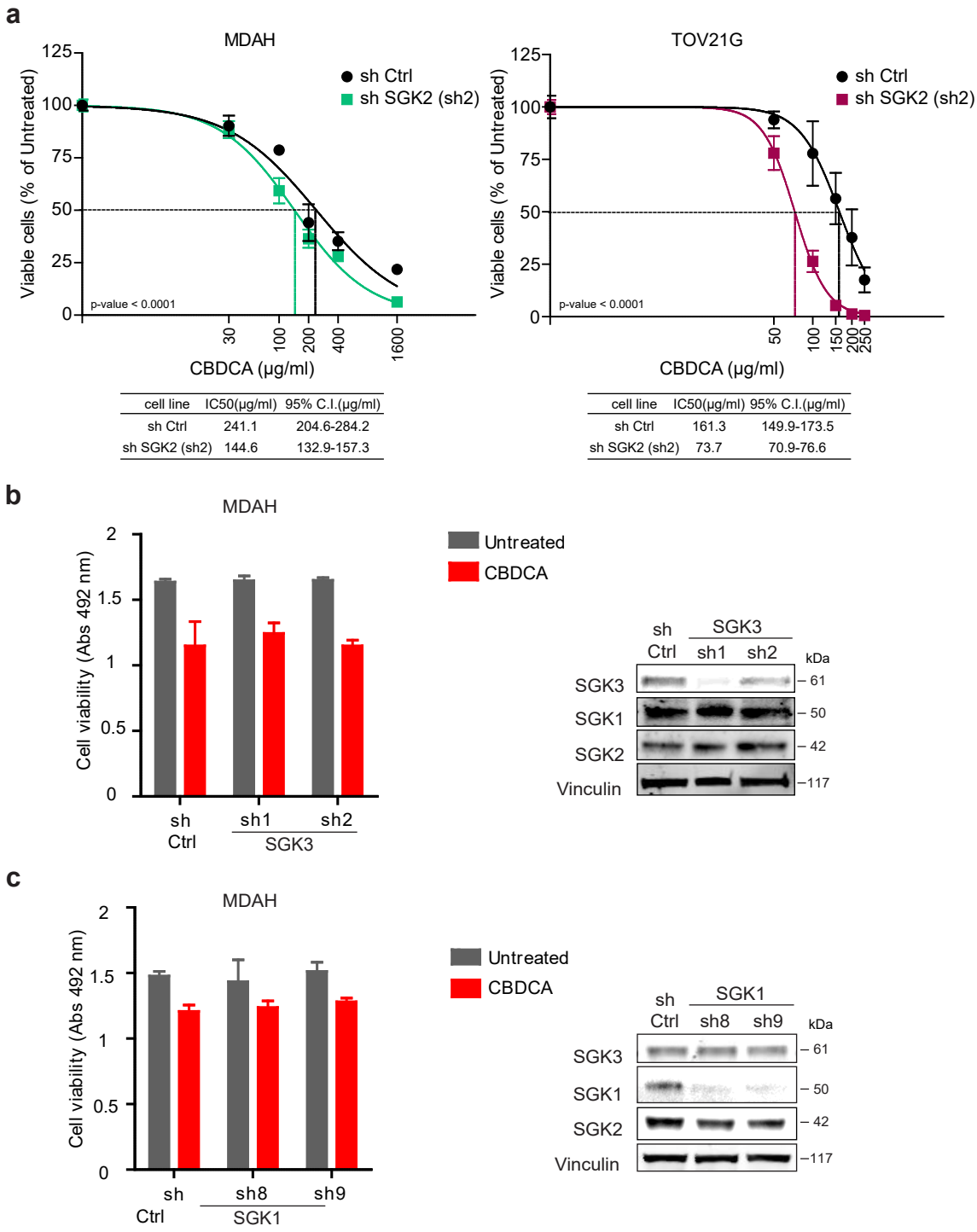

Supplement: Supplementary file 5 — FigureS2 [file 41388_2020_1433_MOESM5_ESM.pdf]

**Fig. S3 (related to Fig. 2)**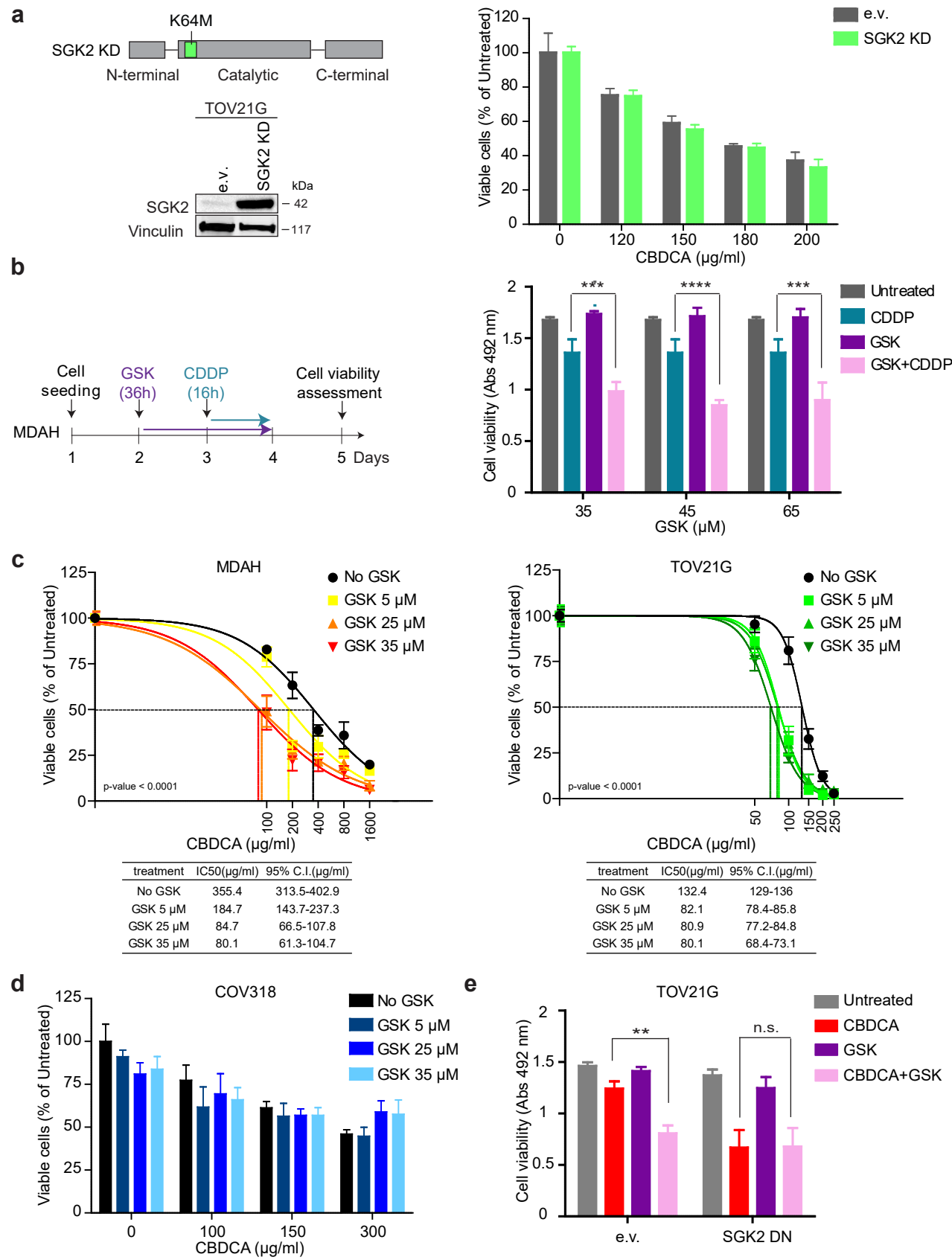

Supplement: Supplementary file 6 — Figure S3 [file 41388_2020_1433_MOESM6_ESM.pdf]

**Fig. S5 (related to Fig. 4)**

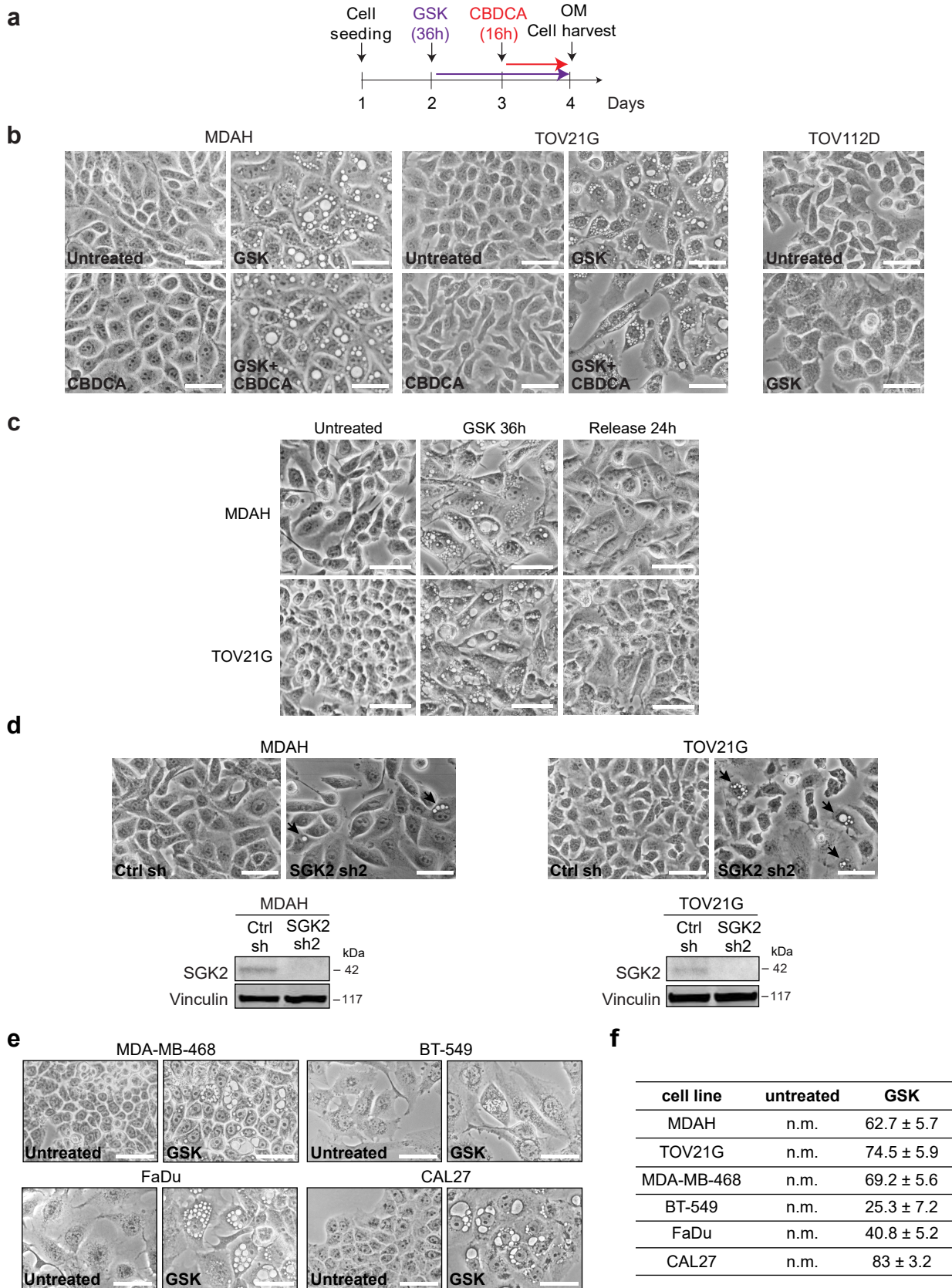

Supplement: Supplementary file 8 — Figure S5 [file 41388_2020_1433_MOESM8_ESM.pdf]

**Fig. S6 (related to Fig. 4)**

**a**

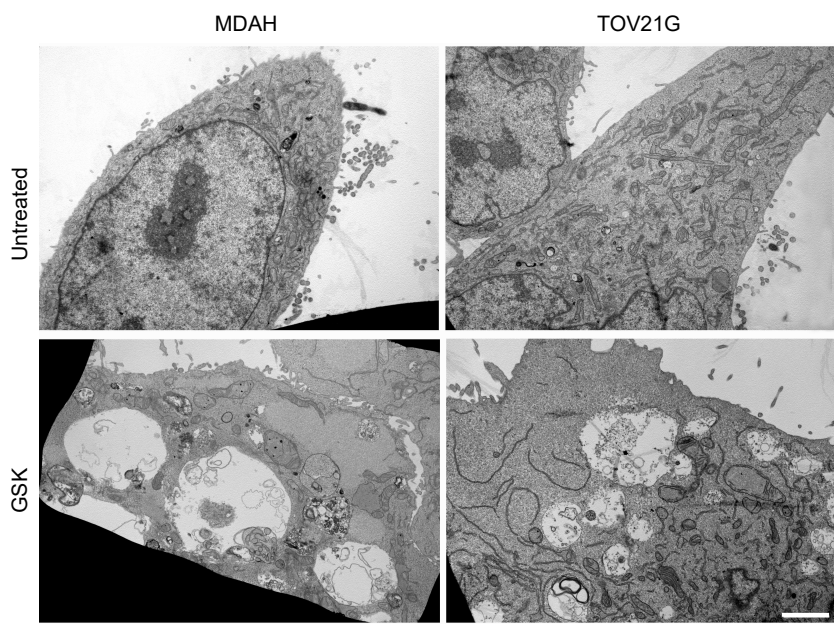

**b**

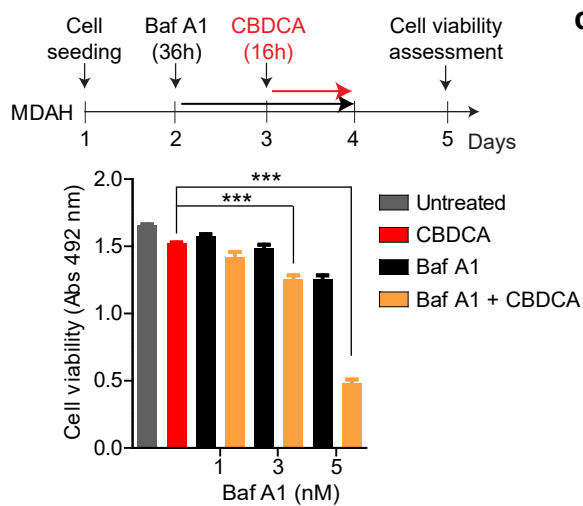

**c**

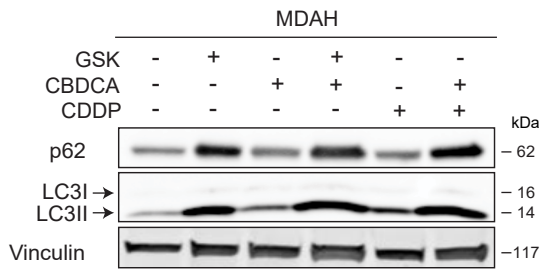

**d**

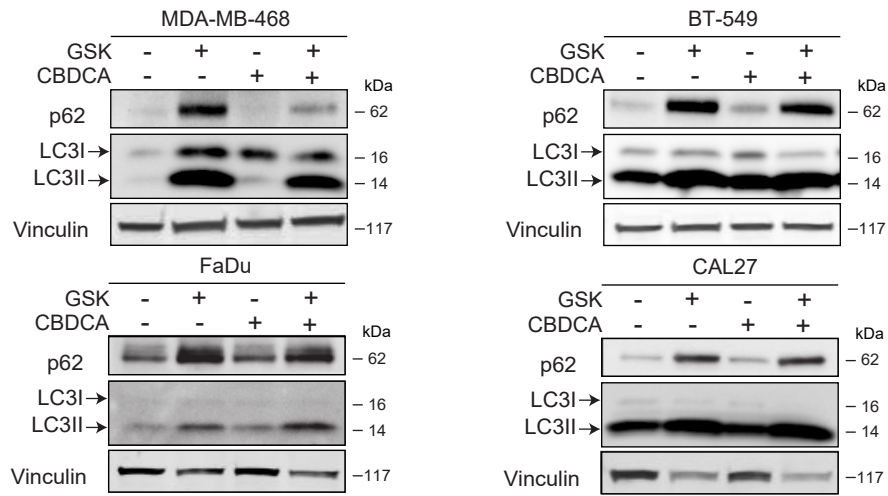

Supplement: Supplementary file 9 — Figure S6 [file 41388_2020_1433_MOESM9_ESM.pdf]

**Fig. S7 (related to Fig. 6)**

**a**

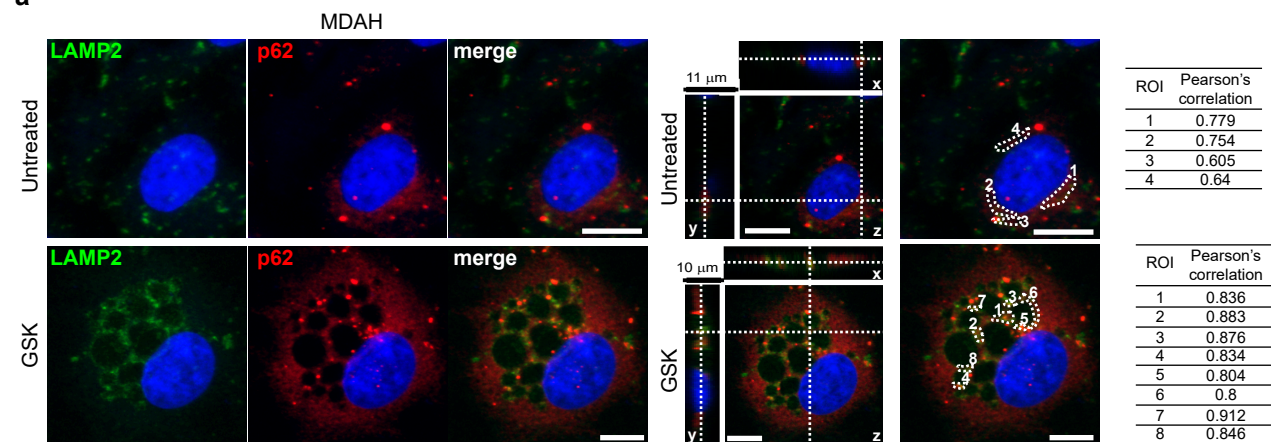

**b**

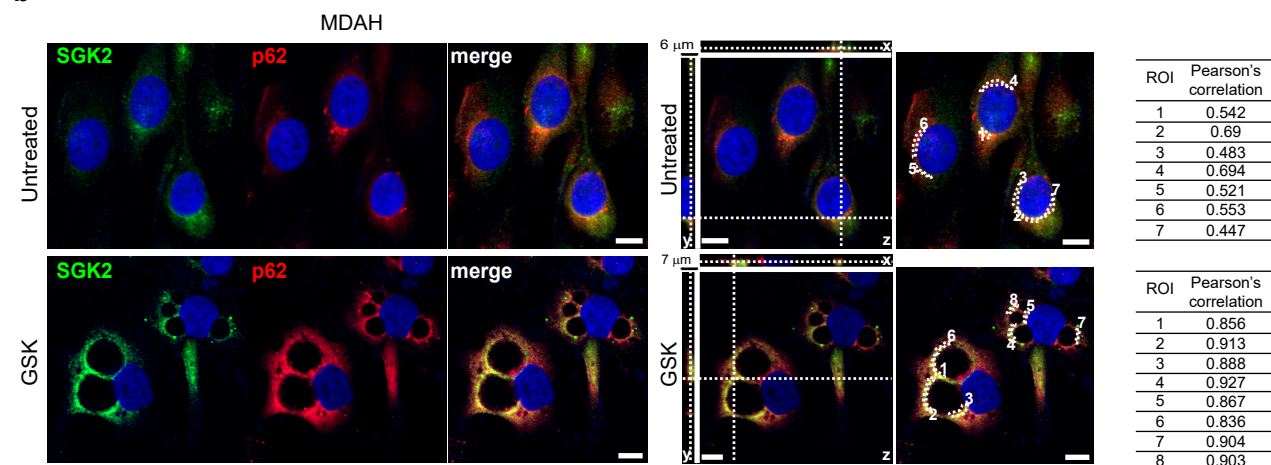

Supplement: Supplementary file 10 — Figure S7 [file 41388_2020_1433_MOESM10_ESM.pdf]

**Fig. S9 (related to Fig. 8)**

**a**

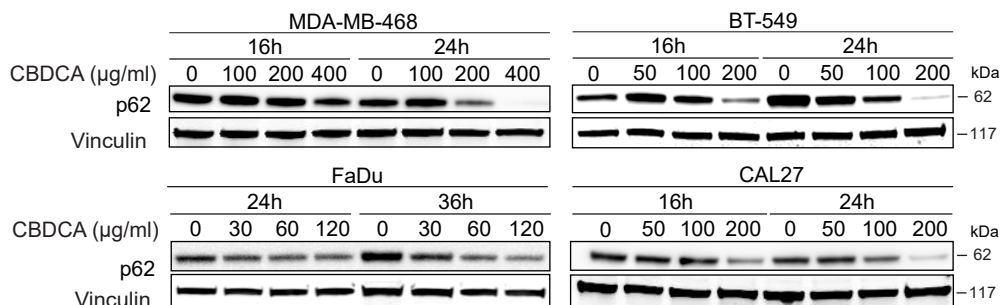

**b**

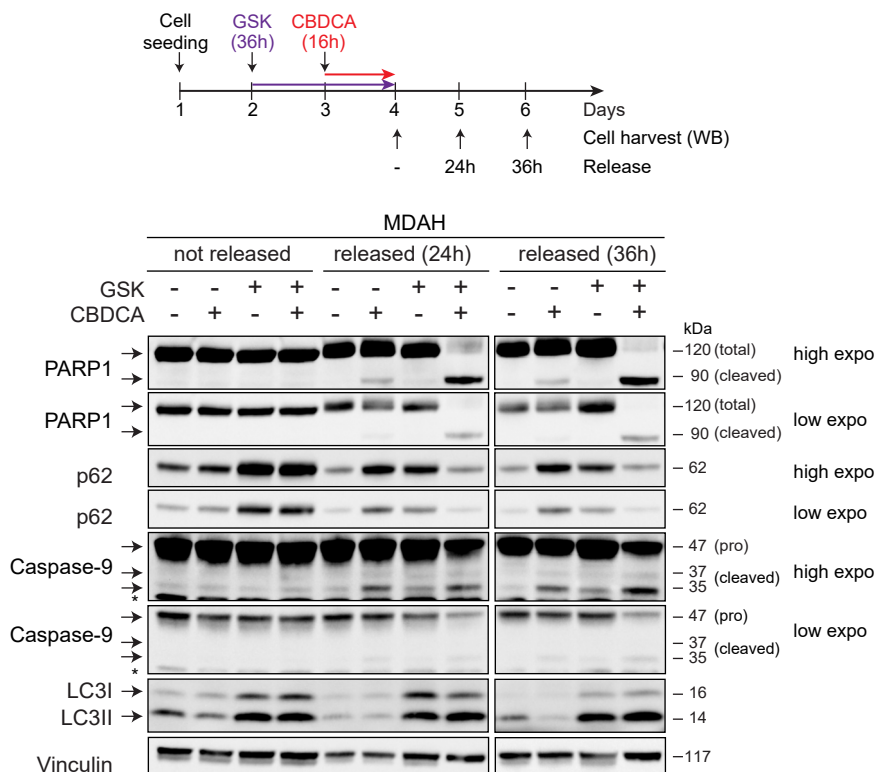

Supplement: Supplementary file 12 — Figure S9 [file 41388_2020_1433_MOESM12_ESM.pdf]

**Fig. S10 (related to Fig. 8)**

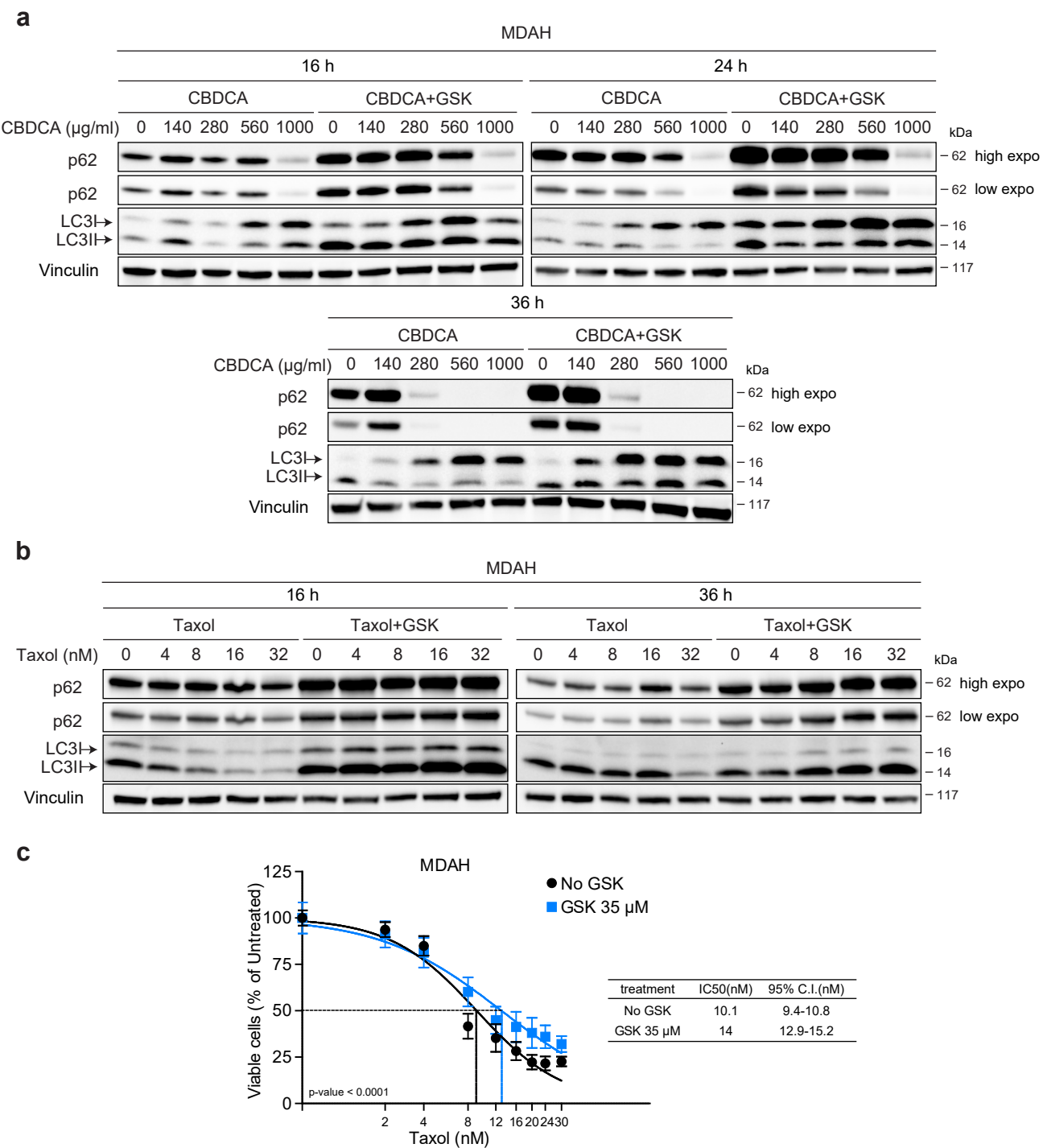

Supplement: Supplementary file 13 — Figure S10 [file 41388_2020_1433_MOESM13_ESM.pdf]

**Fig. S11 (related to Fig. 8)**

**a**

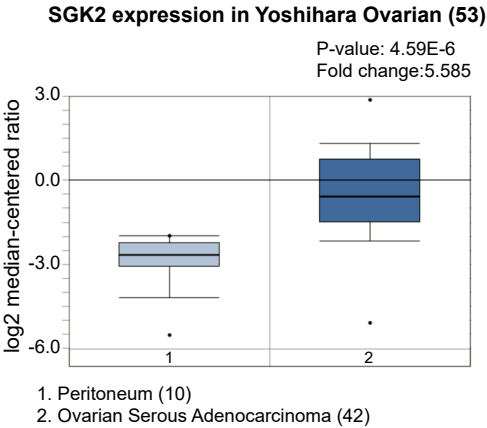

**b**

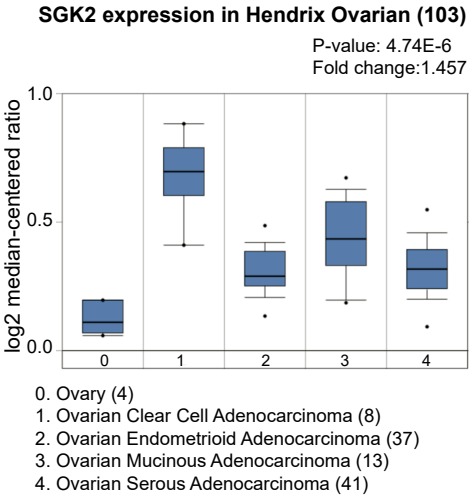

**c**

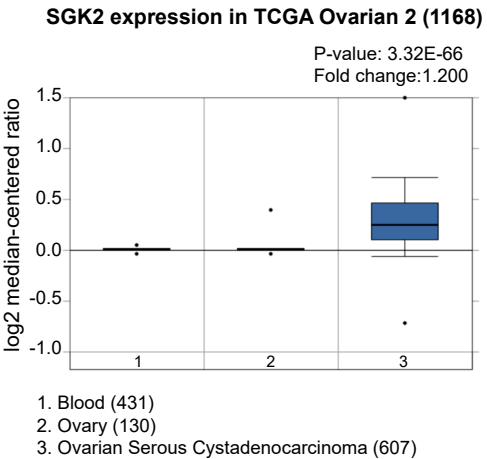

Supplement: Supplementary file 14 — Figure S11 [file 41388_2020_1433_MOESM14_ESM.pdf]
