# Supplementary material for: Serum- and glucocorticoid- inducible kinase 2, SGK2, is a novel autophagy regulator and modulates platinum drugs response in cancer cells
Source: Oncogene. 2020 Aug 27;39(40):6370–86. doi: 10.1038/s41388-020-01433-6 (PMC7529585; doi:10.1038/s41388-020-01433-6)
Supplement: Supplementary file 7 — Figure S4 [file 41388_2020_1433_MOESM7_ESM.pdf]

**Fig. S4 (related to Fig. 3)**

| Number | Tumour | Histotype | Grade | CT/Neoadjuvant | TNM |
|---|---|---|---|---|---|
| 49d | Primary | Clear Cell | G3 | - | pT2bN0 |
| 66 | Recurrence ascites | Serous | n.a. | - | - |
| 77 | Primary | Serous | n.a. | Yes | pT3c |
| 91a | Primary | Serous | n.a. | - | - |
