# Supplementary material for: Serum- and glucocorticoid- inducible kinase 2, SGK2, is a novel autophagy regulator and modulates platinum drugs response in cancer cells
Source: Oncogene. 2020 Aug 27;39(40):6370–86. doi: 10.1038/s41388-020-01433-6 (PMC7529585; doi:10.1038/s41388-020-01433-6)

Fig. S8 (related to Fig. 7)

a

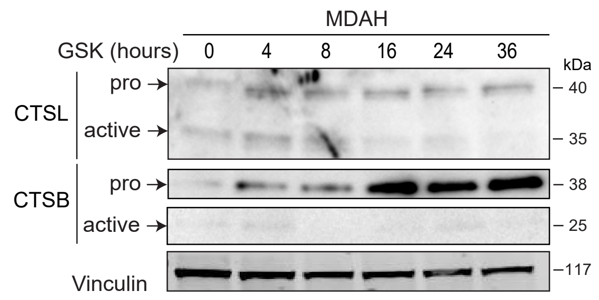

b

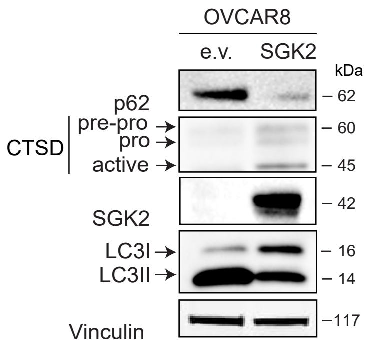

c

V-ATPase subunit	Predicted phosphorylation sites for SGK2	Kinase Predictor V2 Score
ATP6V1H	S174	250
	S180	345
	S190	287
	S192	252
	S194	295
	S351	332
	S354	274
ATP6V0A3	S279	430
	T360	136
	S370	294
	S470	515
	S532	402
	S621	298

d

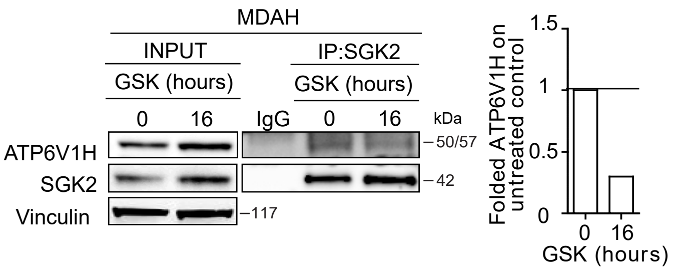

e

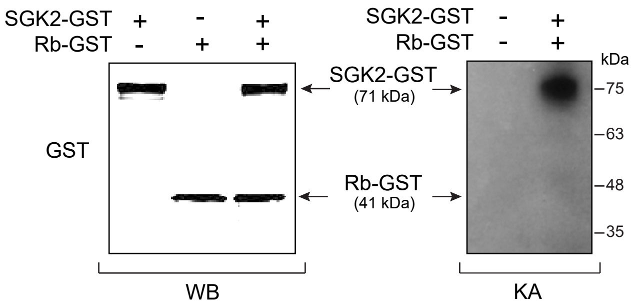

Supplement: Supplementary file 11 — Figure S8 [file 41388_2020_1433_MOESM11_ESM.pdf]
